# A Multiscale *In-Situ* Experimental Method for Characterizing the Mechanical Response of Oil Paint

**DOI:** 10.1007/s11340-026-01297-z

**Published:** 2026-04-15

**Authors:** A. B. Behboud, J. P. M. Hoefnagels, S. Maraghechi, J. J. Hermans, E. Bosco

**Affiliations:** 1 Department of the Built Environment, Eindhoven University of Technology, P.O. Box 513, 5600 MB Eindhoven, The Netherlands; 2 Department of Mechanical Engineering, Eindhoven University of Technology, P.O. Box 513, 5600 MB Eindhoven, The Netherlands; 3 Conservation Science and Chemistry, University of Amsterdam, P.O. Box 94552, 1090 GN Amsterdam, The Netherlands

**Keywords:** Oil paint, *In-situ* tensile testing, DIC, Mechanical properties, Cultural heritage, Paintings

## Abstract

**Background:**

The mechanical behavior of historical oil paint is governed by complex, multiscale chemo-mechanical processes that evolve over decades. Yet, existing mechanical testing approaches cannot fully characterize its tensile response or resolve the microscale deformation and damage phenomena that drive cracking and delamination in historical artworks.

**Objective:**

The aim of this work is to develop an experimental method capable of capturing the mechanical response of small-sized paint samples and linking local deformation and damage mechanisms to their overall tensile behavior.

**Methods:**

A novel methodology is introduced that combines *in-situ* micro-tensile testing and high-magnification optical profilometry with full-field Digital Image Correlation for the first time in oil paint research. The method enables the construction of multiple true stress–true strain curves and the extraction of mesoscopic and macroscopic mechanical properties from a single experiment, allowing statistically meaningful characterization from minimal paint material.

**Results:**

The mesoscale and macroscopic responses provide key mechanical properties (Young’s modulus, yield strength, ultimate tensile strength, strain at break, and Poisson’s ratio) consistent with values reported in the literature. Full-field measurements reveal strain localization near pigment-rich regions, where damage accumulates and microcracks initiate. The tested aged samples consistently exhibit increased stiffness and brittleness, while differences in chemical formulation (Prussian Blue versus Titanium White pigments) lead to distinct ductile or brittle responses.

**Conclusions:**

The proposed methodology enables comprehensive multiscale mechanical characterization of oil paint and captures the deformation mechanisms underlying aging and composition-dependent behavior, representing an important step toward the mechanical characterization of original historical paint material.

## Introduction

Historical oil paintings represent an invaluable part of our cultural heritage. Beyond their artistic significance, from an engineering point of view, they are complex systems composed of multiple oil paint layers, typically applied over a glue-sized canvas and mounted on a wooden stretcher.

**Fig. 1:**
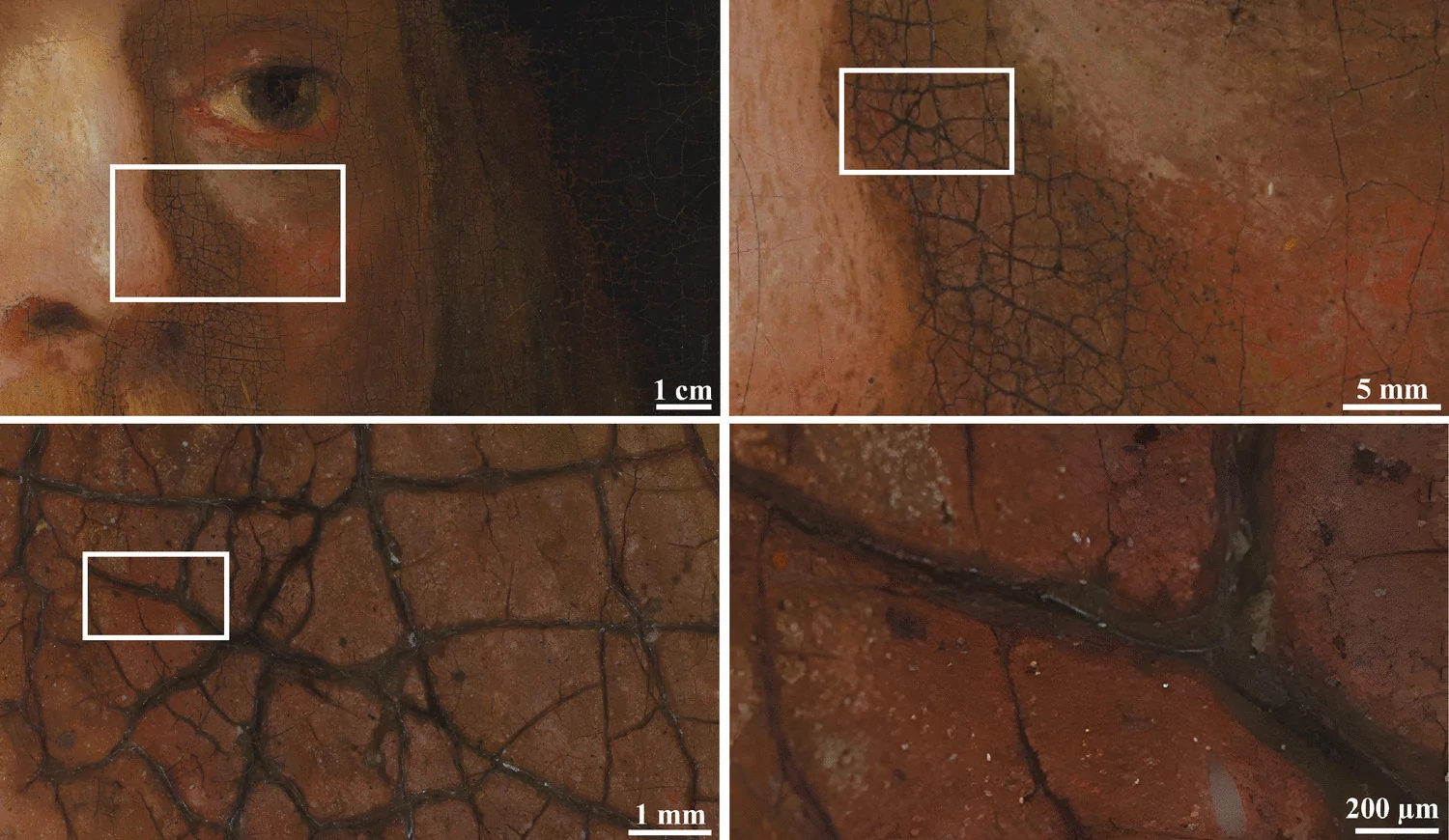
Ultra high resolution images of *The Night Watch* by Rembrandt van Rijn (1642), obtained from the Rijksmuseum digital archive [25]. Multiple cracks in the paint layer are visible. The rectangles in the first three images indicate the regions shown at higher magnification in the subsequent images

Focusing on the oil paint itself, it generally consists of a drying oil binding medium containing dispersed pigment particles. Chemically, drying oils are triglycerides, composed of three fatty acid molecules bound to a glycerol backbone via an ester bond. In the early stages after application, oil paint undergoes curing through autoxidation and polymerization of the drying oils, forming a densely cross-linked polymer network [1–3]. Over longer time scales, oil paint continues to experience a range of complex chemical reactions, including continued oxidation, hydrolysis, and metal soap formation [1, 4–10]. These reactions involve a variety of processes, e.g., bond scission, the formation of new cross-links, and the precipitation of new solid phases, which can induce heterogeneous chemical changes and may contribute to degradation in paint layers at multiple length scales. The extent of these changes depends strongly on the original paint formulation, pigment type, and environmental history of the artwork.

As the chemical state of the paint evolves, so do its mechanical properties. The mechanical behavior of oil paint is typically influenced by material composition, pigment-to-binder ratio, environmental conditions (temperature and humidity), and the age of the paint. Moreover, over time, chemical changes may influence constitutive properties such as strength, ductility, and Young’s modulus [11–15].

Ultimately, the interaction between chemical processes and evolving mechanical response might lead to visible damage, appearing as cracking, delamination, and other failure mechanisms in paint layers [5, 11, 16–24], which are frequently observed in many oil paintings in museum collections. An example of cracking in a historical painting is shown in Fig. 1.

Given the multiscale and time-dependent nature of these chemical and mechanical processes, it is essential to develop versatile, robust, and precise experimental methods to evaluate the mechanical behavior of oil paints at different scales.

In the literature, various experimental techniques have been proposed to mechanically test paint samples, many of which are thoroughly reviewed in [11]. Tensile testing is one of the most commonly used methods for evaluating the global mechanical behavior of (oil) paints through stress–strain curves [6, 13–15, 21, 26–29]. This technique provides a well-defined stress state and yields key mechanical properties such as Young’s modulus, ductility, yield strength, and ultimate tensile strength by constructing visco-elasto-plastic stress-strain curves using global displacement measurement for strain evaluation. However, it is not suitable for obtaining full-field information and requires relatively large specimens, limiting its applicability for historical oil paints. Consequently, it is typically restricted to model system samples; moreover, for strongly aged paints, sufficiently large intact specimens are often difficult to obtain due to their pronounced brittleness and the tendency for extensive cracking during aging, which further limits the feasibility of conventional large-scale testing.

Dynamic Mechanical Analysis (DMA) is another technique suitable for studying visco-elastic materials such as paints [30–33]. It requires a sample size similar to that used in tensile testing and is therefore applicable only to model systems [11]. DMA can provide important material properties such as the glass transition temperature and storage modulus, but it only provides information on the material’s elastic regime, so the plastic properties, including the yield strength, evolution of the flow stress, ultimate tensile strength, or ductility, are not accessible. Moreover, although this technique offers a well-defined stress state, it does not deliver full-field information. Micro- or nanoindentation is one of the most widely used and preferred techniques for studying the mechanical behavior of oil paints [31, 32, 34–38]. Compared to tensile testing, it requires a much smaller sample size, making it suitable for application to historical materials [11]. However, it does not provide well-defined information about the visco-elasto-plastic material behavior of the paint. First, the technique is inherently limited to compressive loading, whereas paint (layers) predominantly fail in tension. Second, although indentation allows localized probing of mechanical properties with high spatial resolution, it is less suitable for highly heterogeneous and strongly pigmented oil paints [37]. The presence of stiffer pigment particles can cause significant local increases in the reduced modulus [11], leading to an overestimation of the elastic modulus (often referred to as the indentation modulus). Third, because the method does not impose a well-defined homogeneous global stress state (in fact, the stress state is highly inhomogeneous), the yield strength and ultimate tensile strength can only be inferred indirectly and are therefore typically reported as the indentation hardness. Finally, the spatial resolution for nanoindentation is lower than the name suggests, as the stress field underneath the tip stretches over micrometers; a large number of indents is usually required to adequately average over the material heterogeneity at the microscale. On the other hand, qualitative property maps can be constructed using high-density indentation grids [36].

**Table 1:**
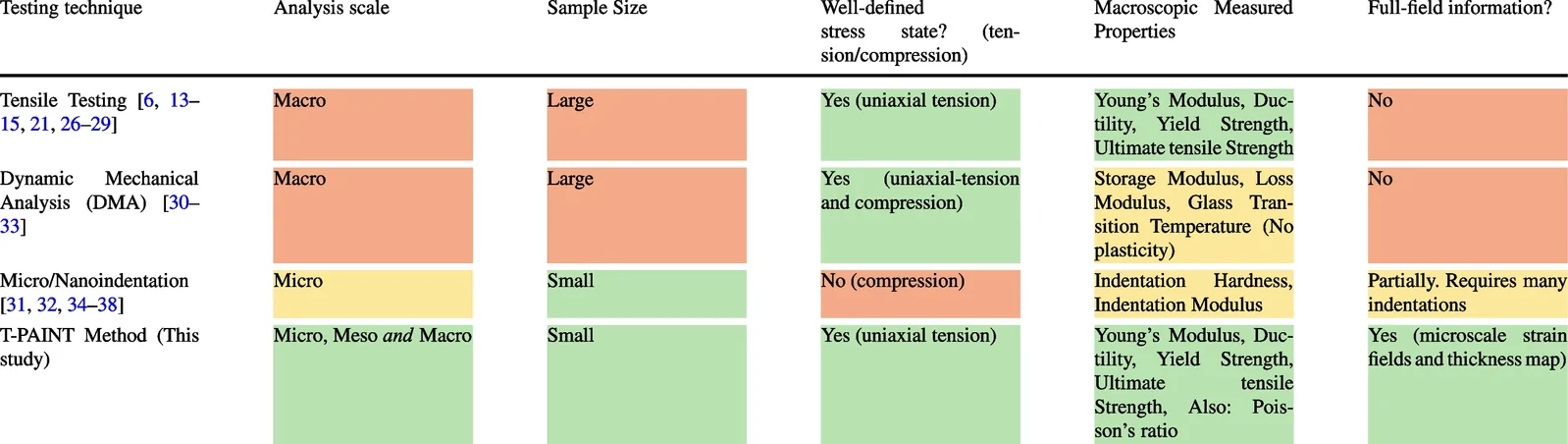
Summary of experimental techniques and their comparison based on five key characteristics. The most relevant literature references for each method are listed. A color code is used to indicate performance: green denotes advantages, red denotes drawbacks, and yellow represents intermediate or moderate performance

In this study, a novel *in-situ*^1^ experimental mechanics methodology, referred to as **T-PAINT** (**T**U/e - **P**olymer/**P**aint **A**nalysis by **IN**-situ micro-**T**ensile experiment), is proposed for investigating the mechanical behavior of oil paint samples. The method builds upon micro-tensile testing combined with full-field Digital Image Correlation (DIC), which appears to be applied to oil paint samples here for the first time. T-PAINT is specifically designed to directly relate paint microstructural and chemical features to mechanical behavior. The framework is applicable to small-scale paint samples and, although currently demonstrated on model systems, represents an important step toward the mechanical characterization of original, historical paint material. Through these combined characteristics, the T-PAINT methodology overcomes several limitations of the existing techniques discussed above. Table 1 summarizes the most commonly used experimental approaches for assessing the mechanical properties of oil paints and positions the T-PAINT method within this landscape.

**Table 2:** Overview of the samples considered in this study

Sample name	Wet thickness (μm)	Age (weeks)	Aging Condition
Titanium White	250	0, 4, 14, 16 weeks (artificially aged)	$50\,^\circ \textrm{C}$ and 75% RH
Prussian Blue	200	4 weeks (artificially aged)	$65\,^\circ \textrm{C}$ and 20-80% RH

The T-PAINT method enables a comprehensive mechanical analysis across different scales: (i) At the *microscale*, accurate geometry measurement is first achieved through high-magnification optical profilometry that provides local thickness, width, and cross-sectional area along the specimen gauge length. Moreover, full-field microscale strain mapping, obtained via DIC, produces spatially resolved strain maps at each loading step and captures heterogeneous deformation, strain localization, and onset of damage. (ii) At the *mesoscale*, the method enables the extraction of multiple mesoscale stress–strain responses from a single experiment. These are evaluated at discrete Regions of Interest (ROIs) by combining the axial strain averaged over each ROI with the corresponding local stress computed from the applied force and the ROI-average of the measured cross-sectional area. Assuming that the material and morphological variations within a paint sample (intra-sample variations) are comparable to those between different samples (inter-sample variations), the ensemble of these local responses can be regarded as additional statistically meaningful mechanical data, complementing the global stress–strain curve. This feature is essential in cultural heritage applications, where only extremely limited quantities of historical paint are available for testing. (iii) At the *macroscale*, the global mechanical behavior is estimated by averaging the microscale strain over the full field of view and combining it with the applied force to construct the macroscopic stress–strain response and global properties such as Young’s modulus, ductility, and ultimate tensile strength.

The method is demonstrated here for two very different oil paint formulations (Titanium White and Prussian Blue) under both unaged and artificially aged conditions. However, the T-PAINT methodology is general and can be extended to other paint systems (e.g., acrylics) without loss of generality.

This paper is organized as follows. Section “T-PAINT Method” presents the methodology, including sample preparation, dimension measurements, tensile testing, and the Digital Image Correlation procedure, together with its specific challenges and the solutions implemented to ensure reliable full-field measurements. Finally, Section “Proof of Principle of the Methodology Performance” demonstrates the performance of the methodology and discusses the results obtained at the macro- (global), meso-, and microscale. Concluding remarks are finally given in Section “Conclusions”.

## T-PAINT Method

### Sample Preparation

The mock-up paint films were prepared by mixing Titanium White and Prussian Blue pigments with cold-pressed linseed oil by an automatic muller. The fresh oil paint was next applied onto a ≈20 μm thick polyester substrate with a film applicator (paint film wet thickness of 250 or 200 μm) to ensure a uniform wet-film thickness. Once the mock-up films reached a touch-dry state, they were subjected to artificial aging in a climate chamber under controlled temperature and humidity for varying durations. The characteristics of the samples and the aging protocols are summarized in Table 2. After aging, samples with dimensions of approximately $4~ \textrm{mm} \times 600$ $\mu \textrm{m}$ were extracted from the mock-up films for testing, using ultra-thin sharp razor blades to minimize the risk of preparation-induced surface damage and microcracks.

**Fig. 2:**
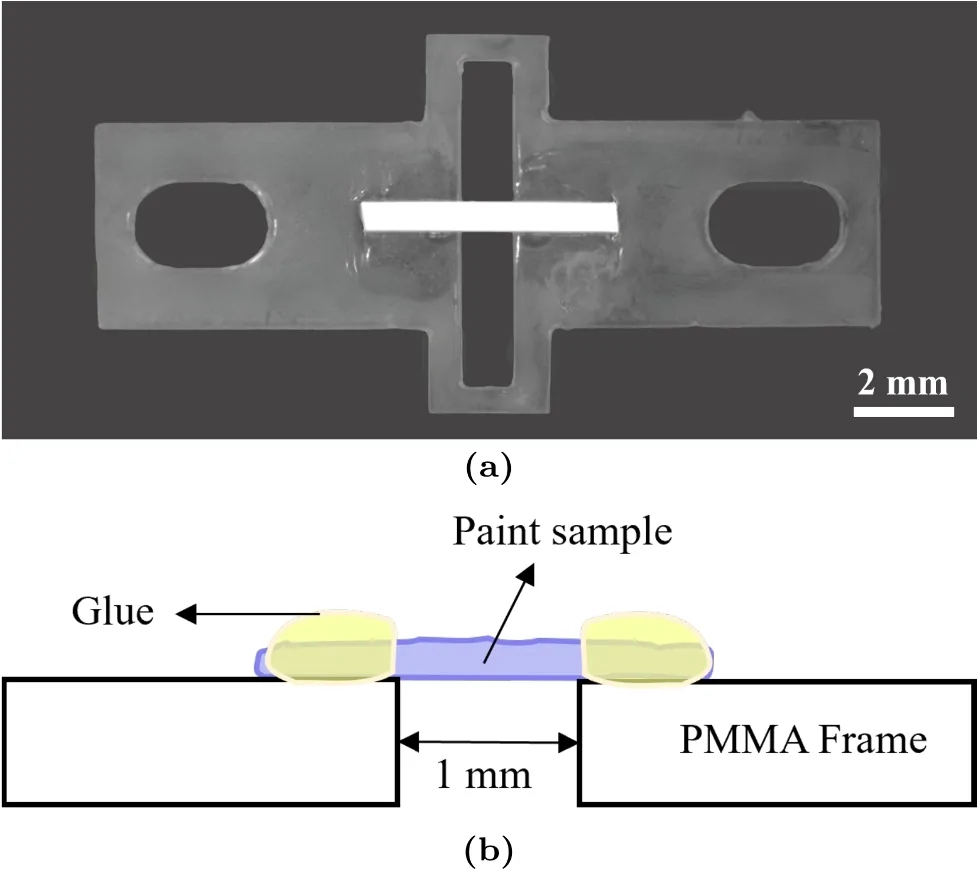
(**a**) A Titanium White oil paint specimen attached to a PMMA frame that maintains the sample integrity before testing. (**b**) Schematic representation of the cross-sectional view

The small-sized paint samples needed to be handled with utmost care; therefore, each specimen was mounted to a small PMMA (Polymethylmethacrylate) frame to avoid damaging them prior to testing. The PMMA frames were cut from a 0.5 mm-thick sheet using a laser cutter, following the construction procedure proposed in [39]. The frame contains a central window with a width of approximately 1 mm, defining the gauge length, and two side bridges with a width of approximately 700 μm; see Fig. 2. Before testing, the two bridges prevent the sample from unintended loading and protect it from irreversible deformation or damage during dimension measurements, speckle pattern application, or placement of the frame in the tensile tester.

Each paint sample was adhered to the frame using nail polish, according to the method of [39], by placing small droplets on both sides and aligning one end of the sample with a fine-tip sticky needle. The opposite end of the sample was then gently pressed onto the droplet to secure the specimen. The adhesive was allowed to cure for at least 24 hours before cross-sectional measurements and DIC patterning. It was observed that the glue (nail polish) never penetrated the gauge section of any sample.

### Dimension Measurements

Accurate measurement of the cross-sectional area of the tensile sample is essential to calculate the stress corresponding to the applied force. This measurement is particularly important because the final dry thickness of the samples was consistently different from their initial wet thickness. This reduction in thickness over time is typical for paint films and can be attributed to the evaporation of organic medium components and low-molecular-weight molecules during the drying and aging processes [15], leading to contraction in the thickness direction.

**Fig. 3:**
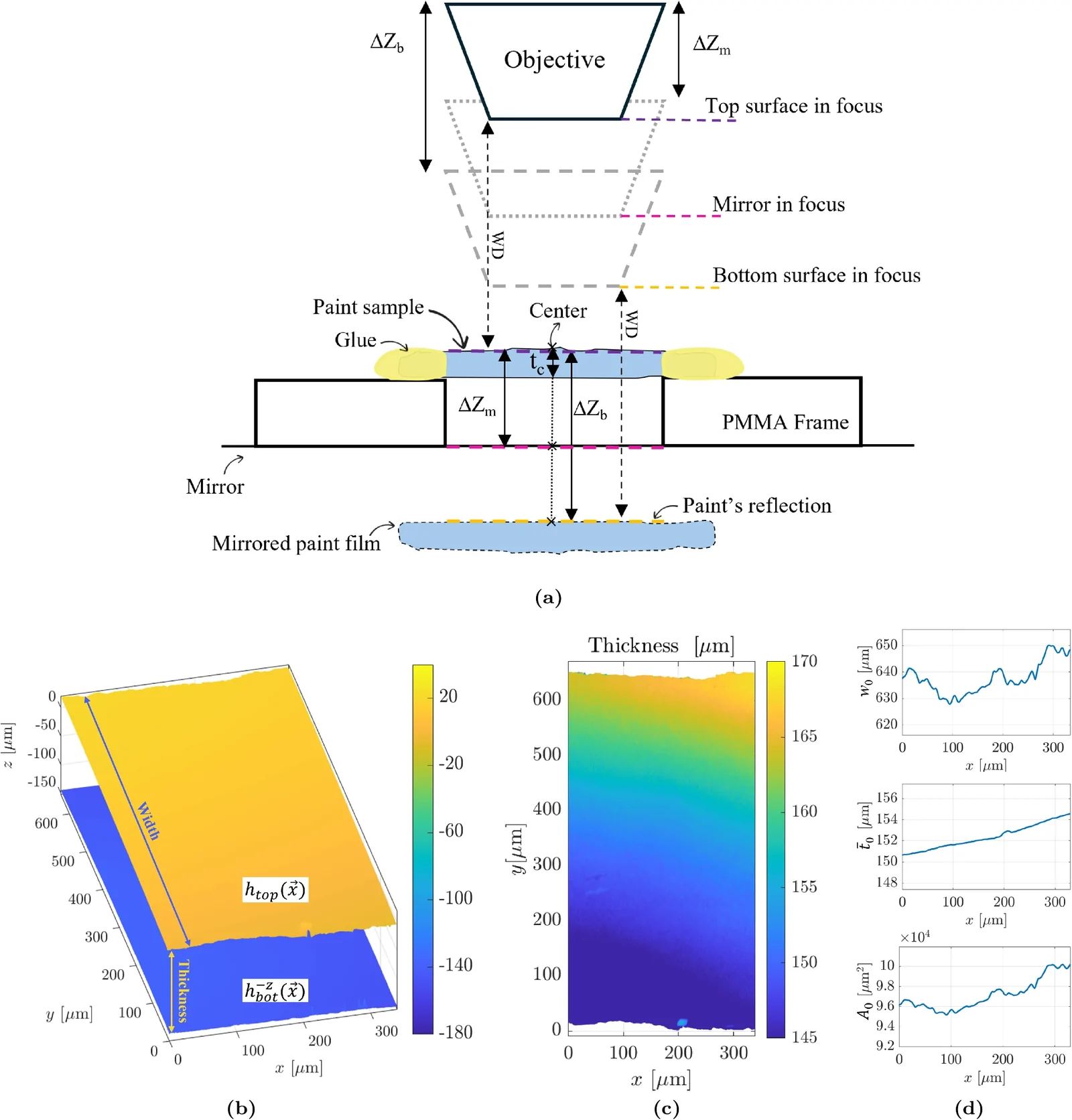
(**a**) Schematic illustration of the cross-sectional measurement procedure using optical profilometry. “WD” refers to the working distance of the objective. Example measurements from one representative paint sample: (**b**) Top and bottom surface height profiles positioned at the local thickness distance to visualize thickness and width. (**c**) Reconstructed two-dimensional thickness profile of the sample. (**d**) Width ${w}_0(x)$, average thickness $\bar{t}_0(x)$, and cross-sectional area ${A}_0(x)$ profiles measured along the *x*-direction of the paint sample

To measure the width and thickness of the samples with high precision, a method based on optical profilometry is employed, following [39, 40]. A confocal optical microscope (Sensofar S neox) is used to measure the height profiles of the paint sample surfaces. A 50× super-long working distance objective lens provides a sufficiently large clearance above the PMMA frame, enabling a more efficient measurement process compared to previous approaches [39–41]. The complete thickness measurement procedure is described below.

**Fig. 4:**
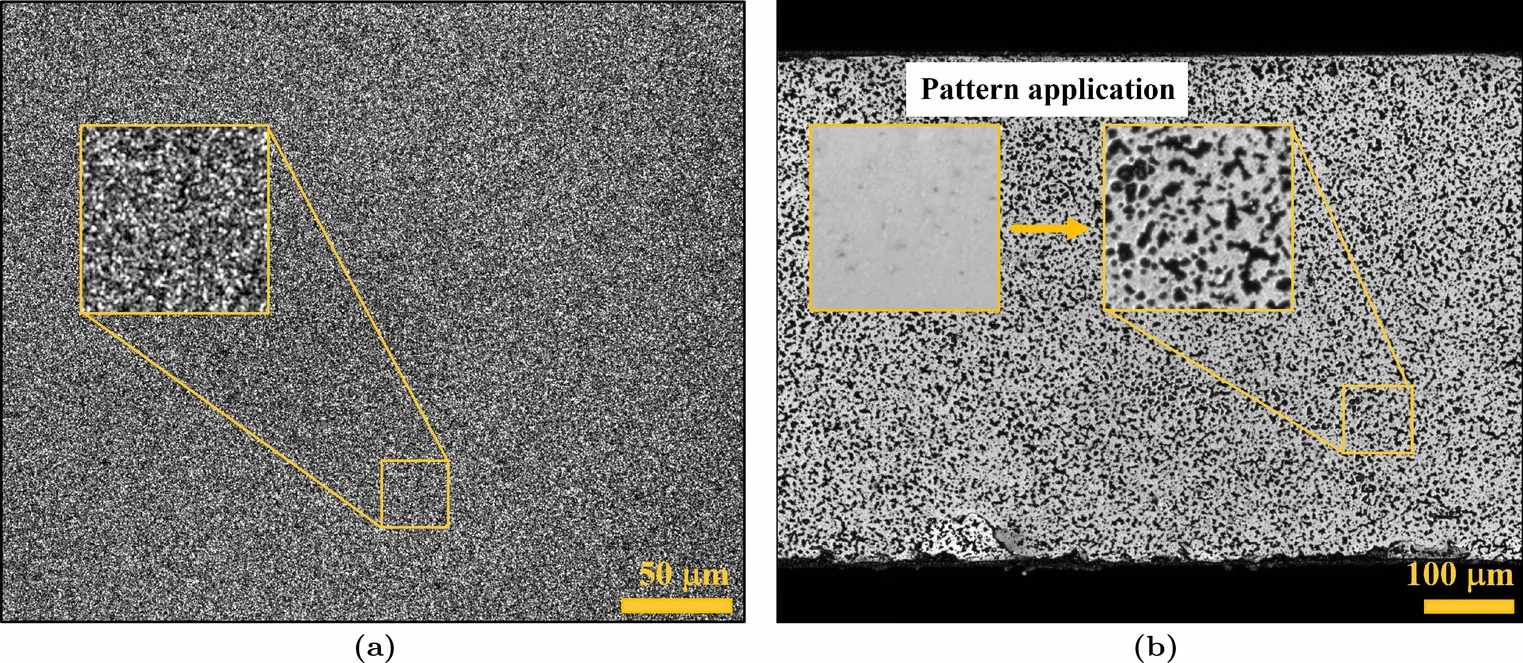
Optical profilometry images of (**a**) the natural speckle pattern with high average image brightness of the surface of a Titanium White oil paint sample, and (**b**) the applied speckle pattern produced by airbrushing polystyrene particles (black dots) onto the top surface (bright background) of a Prussian Blue oil paint sample

Before tensile testing, the PMMA frame to which the paint specimen is attached is placed on top of an optical mirror (i.e., a silicon wafer), as shown in Fig. 3(a). The top surface of the sample is first imaged to obtain the height profile $h_{top}(\vec {x})$, with $\vec {x} = (x,y)$, constructed from five overlapping scans across the sample width. As illustrated in Fig. 3(a), the optical system is then moved downward to focus on the mirror surface beneath the sample, and the corresponding moving distance $\Delta Z_m (\vec {x})$ (i.e., the travel distance of the microscope focus from the top surface to the mirror) is recorded. This distance represents the sum of the thickness of the paint sample $t_0(\vec {x})$ and that of the PMMA frame. Here, the subscript 0 emphasizes that the thickness measurement is performed in the undeformed configuration. Because the exact frame thickness is not known a priori due to laser-cutting irregularities, the optical system is subsequently adjusted to focus on the reflection of the bottom surface of the paint sample in the mirror. This allows acquisition of the height profile of the bottom surface $h_{bot}(\vec {x})$ and provides the corresponding moving distance $\Delta Z_b(\vec {x})$ (i.e., the travel distance of the microscope focus from the top surface to the reflection of the bottom surface in the mirror). Note that the moving distances $\Delta Z_m(\vec {x})$ and $\Delta Z_b(\vec {x})$ depend on the in-plane position on the sample surface.

In the post-processing stage, a point with identical in-plane coordinates on both the top and bottom surfaces of the sample is selected as the reference (the *center point* $\vec {x}_c$), and both height profiles are vertically shifted to be zero at the center point, i.e., $h_{top}(\vec {x}_c)=0$ and $h_{bot}(\vec {x}_c)=0$. Then, the local thickness of the paint sample at center point is calculated from the measured focus-shift distances as:

1 $$\begin{aligned} t_c=t_0(\vec {x}_c) = 2 \Delta Z_m(\vec {x_c}) - \Delta Z_b(\vec {x_c})\ ~. \end{aligned}$$

The bottom surface height profile is then digitally mirrored with respect to the x-y plane (i.e., the z coordinate is reversed) and subsequently shifted downward by $t_c$ as shown mathematically in Equation (2) and visually in Fig. 3(b):

2 $$\begin{aligned} h_{bot}^{-z}(\vec {x})=-h_{bot}(\vec {x})-t_c~. \end{aligned}$$

Finally, the thickness profile of the sample is determined as:

3 $$\begin{aligned} t_0(\vec {x}) = h_{top}(\vec {x}) - h_{bot}^{-z}(\vec {x}). \end{aligned}$$

An example of a reconstructed thickness profile for an oil paint sample is illustrated in Fig. 3(c).

The thickness profile of the sample in the longitudinal direction $\bar{t}_0(x)$ is determined by averaging the thickness ${t}_0(\vec {x})$ across the sample width, with the width ${w}_0(x)$ obtained directly from the height profile of the top surface (Fig. 3(b), (c)). The cross-sectional area of the sample in the longitudinal direction ${A}_0(x)$ is finally calculated as the product of the local width ${w}_0(x)$ and thickness $\bar{t}_0(x)$, as illustrated in Fig. 3(d). In cases where the height profiles of the bottom surface have a few missing data points due to limited optical signals from the reflection, a standard cubic spline interpolation is used.

**Fig. 5:**
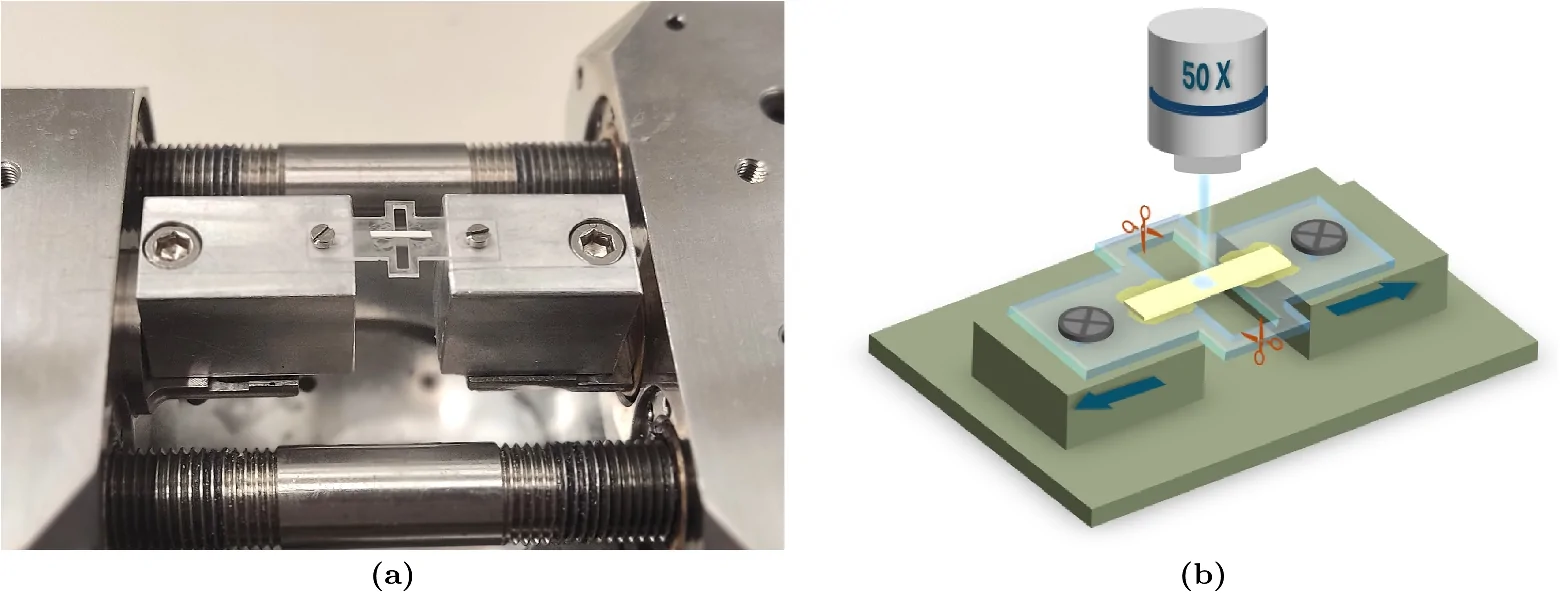
(**a**) Oil paint sample attached to the PMMA frame mounted on the micro-tensile stage. (**b**) Schematic of the *in-situ* test setup consisting of the micro-tensile stage under the 50× objective

### DIC Speckle Pattern Application

To perform DIC (see Section “DIC Analysis”), a fine and stable surface pattern with a high average brightness gradient is required [39, 42]. This pattern should provide the gray-scale contrast needed to reliably track and evaluate deformation during *in-situ* tensile experiments. When the natural surface of a sample does not provide sufficient gray-scale variation, an external pattern needs to be applied. A suitable pattern must be sufficiently random, exhibit high contrast, and adhere well to the sample surface to remain stable and provide an optimal average image gradient [42–44].

The quality of a speckle pattern strongly depends on the surface characteristics of the specimen. Materials with smooth surfaces, such as metals and ceramics, typically accept spray or paint-based patterns well, although surface preparation (cleaning or polishing) may be needed. Polymers often exhibit natural surface textures that can interfere with pattern uniformity and may require more specialized application techniques. Oil paints introduce additional complexity due to their heterogeneous and pigment-rich surfaces, and different samples may require different approaches to obtain a reliable pattern. In this study, two types of oil paint samples (Titanium White and Prussian Blue) were examined, each with distinct surface properties and aging durations - see Table 2. Accordingly, both natural and externally applied speckle patterns are used, depending on the sample type.

For Titanium White samples, the intrinsic surface morphology of the oil paints provides sufficient natural gray scale contrast, making external patterning unnecessary. An example of such a natural surface pattern is shown in Fig. 4(a). It is important to verify that this natural contrast remains stable during loading; this aspect is further examined in Section “Micromechanical Test and Real-Time Full-Field Observation”.

For Prussian Blue samples, the natural surface contrast is insufficient, and an external speckle pattern is therefore applied. In this case, a three-dimensional speckle pattern is generated using a diluted solution prepared at a concentration of 10 μL of 1-μm-diameter spherical polystyrene particle solution (concentration of stock solution is 50 mg/mL) per 1 mL of ethanol and sonicated in a cold water bath for 30 minutes to break apart particle clusters. The solution is then sprayed on the sample from a 13 cm distance for approximately 40 minutes, using an airbrush with 1 bar pressure. Airbrushing from a relatively large distance allows fine control over speckle size and density, making this approach suitable for small-scale or delicate specimens. Note that the speckle pattern is deposited as a single, relatively sparse surface layer. Consequently, the increase in sample thickness due to patterning is minimal (well below 1 % of the total sample thickness) and has a negligible effect on the effective cross-sectional area. An example of the resulting micro-polystyrene speckle pattern is shown in Fig. 4(b).

**Fig. 6:**
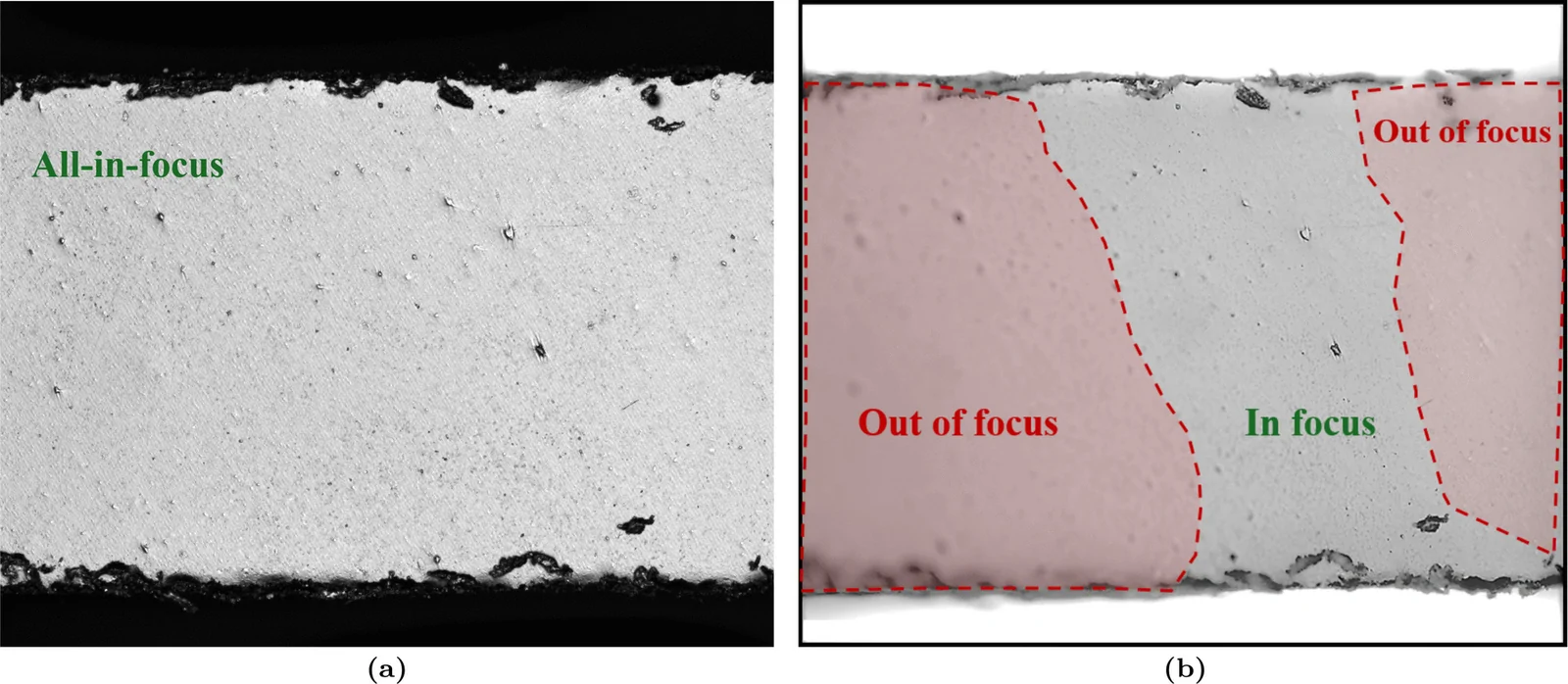
(**a**)“All-in-focus” optical profilometry image of an oil paint sample. (**b**) Optical light microscopy image of the same oil paint sample, showing that not all regions are in focus

### Micromechanical Test and Real-Time Full-Field Observation

After the oil paint sample is attached to the PMMA frame and the speckle pattern is applied, the *in-situ* uniaxial tensile test is performed. For this purpose, a micro-tensile stage (Kammrath & Weiss) with a 10 N load cell is used (Fig. 5(a)). Real-time image acquisition is carried out using an optical profilometer (Sensofar S neox) with a 50× objective lens, as shown schematically in Fig. 5(b). Optical profilometry provides high-resolution, “all-in-focus” images (see Fig. 6(a)). This is not achievable for a regular optical microscope (see Fig. 6(b)) due to the surface roughness of the sample, typically approximately 15 μm and with even larger local peaks. Moreover, during tensile loading, out-of-plane movements on the order of 30-50 μm can occur. Because profilometry images are inherently independent of out-of-plane motion, it is not necessary to use stereo-DIC, which requires two cameras to track the surface in 3D and, consequently, offers a significantly lower maximum spatial resolution [45]. Additionally, the optical profilometry is a vertical scanning microscope, which allows automatic height adjustments during the tensile test.

To enable imaging with a short working distance objective, two aluminum blocks are mounted on the grips of the tensile stage, and the PMMA frame is screwed onto these blocks, following [39, 40] - see Fig. 5(a). Before tightening the screws, the sample is carefully aligned with the loading direction. The paint sample on the frame is examined under low magnification to verify that it is oriented correctly with respect to the loading axis. If any misalignment is observed, the frame is gently rotated until proper alignment is achieved. Once the sample is aligned, the screws are tightened, and the two side bridges of the PMMA frame are delicately melted using a soldering iron (Fig. 5(b)) to ensure full transfer of the tensile load to the sample during testing.

**Fig. 7:**
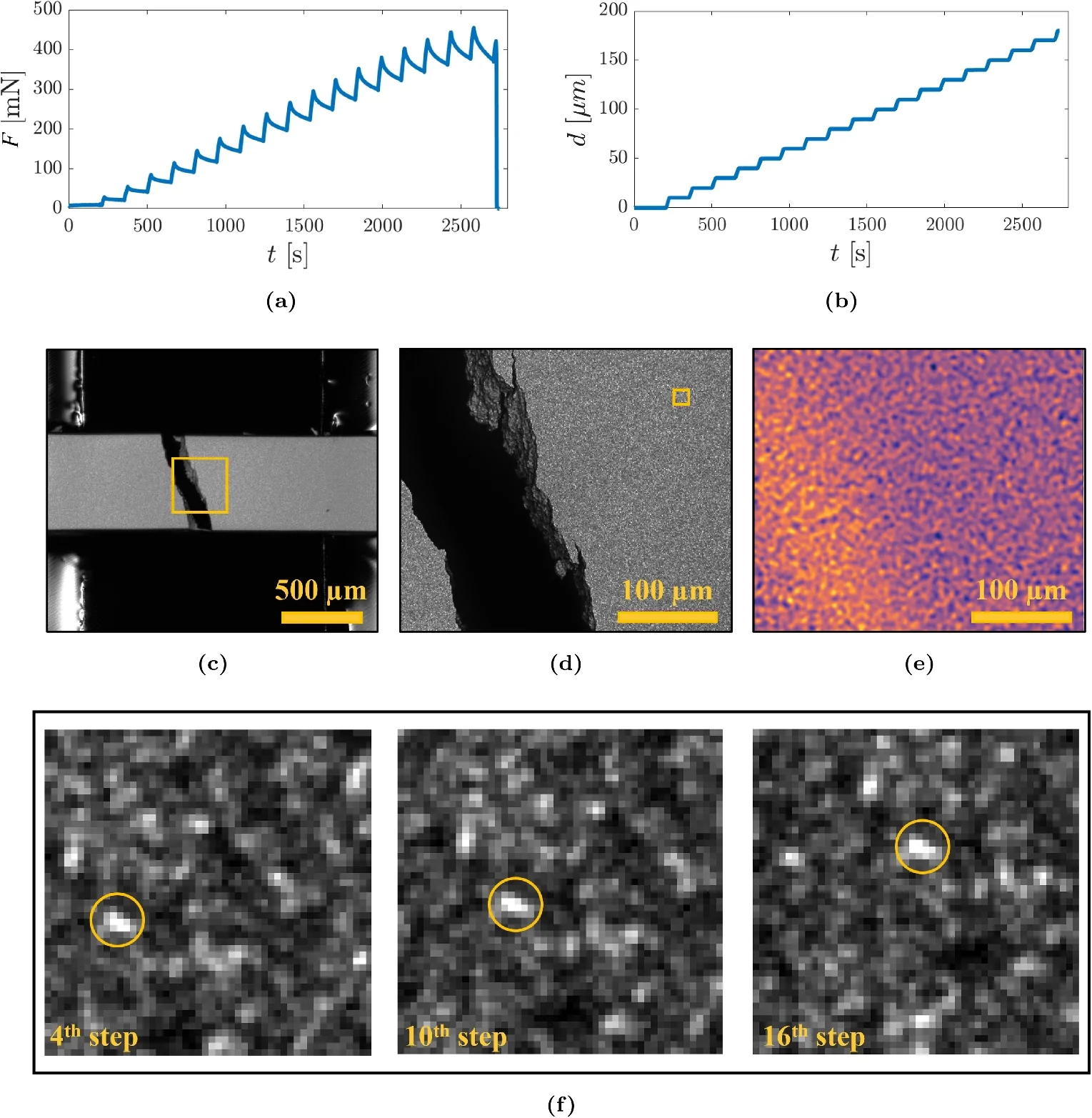
Stepwise micro-tensile test results of a representative Titanium White sample: (**a**) Force *F* as a function of time *t*. (**b**) Displacement *d* as a function of time *t*. (**c**) Fracture at 5× magnification. (**d**) Detailed view of the fractured area at 50× magnification; the yellow square shows the region where the image series in (**f**) is acquired. (**e**) Map of the strain component $\varepsilon _{xy}$ obtained from DIC analysis of the last image before fracture. The strain map corresponds exactly to the same area as (**d**). (**f**) Stepwise image series showing tracked feature

The micro-tensile test is performed in a stepwise, displacement-controlled manner at a speed of 0.5 μm/s, corresponding to a strain rate of approximately $5 \times 10^{-4}$ $\,\textrm{s}^{-1}$. Relatively small, fixed displacement increments are applied, with the corresponding force value measured by the load cell. In addition to monotonic loading, selected tests include unloading-reloading cycles, which provide insight into the visco-elasto-plastic behavior of the paint films. At each loading step (including the initial undeformed configuration), an image is acquired from the central region of the sample with a field of view of approximately 340 μm × 280 μm. The stepwise application of the load and image acquisition continue until the rupture of the paint sample. The acquisition of each image takes about 2 minutes, during which some degree of stress relaxation occurs due to the visco-elastic behavior of the paint (Fig. 7(a) and (b)). The force value stored for each loading step corresponds to the relaxed force value obtained at the end of the imaging period.

Figure 7(f) shows a 50×50 pixels zoomed sequence of images captured at successive loading steps during a tensile test on a Titanium White oil paint sample. The tracked feature (indicated with yellow circles) gradually changes location on the surface of the sample as a result of the small accumulated deformations. Comparison of the images confirms that the surface contrast remains stable across loading steps, with a negligible difference in average contrast and brightness. This demonstrates that the natural surface pattern of Titanium White samples remains consistent during testing and is therefore suitable for reliable DIC analyses.

Figure 7(c) and (d) show the Titanium White oil paint sample after fracture, imaged by the optical profilometer at 5× and 50× magnification, respectively. After completion of the *in-situ* micro-tensile test and acquisition of the full image sequence, DIC is employed to obtain the displacement and strain fields. The details of this procedure are elaborated in Section “DIC Analysis”. Figure 7(e) illustrates the strain map of the component $\varepsilon _{xy}$ obtained from the final image before fracture. Comparison of Fig. 7(d) and (e) clearly indicates that strain localizes precisely in the region where fracture ultimately occurs, and the orientation of strain localization in Fig. 7(e) matches exactly the orientation of the crack in Fig. 7(d).

### DIC Analysis

#### Theory

In the final stage of the T-PAINT method, the images of the paint samples are analyzed using local DIC with optimized settings to extract the local displacements and strains at each loading step. In local DIC, the zone of interest (ZOI) in an image is divided into many small subsets Ω, typically square blocks. For each subset, the algorithm searches the deformed image for the location that best matches the reference subset, while allowing for simple deformation modes (e.g., affine deformation). The displacement vector is then calculated for the center of the subset [43, 46]. Contrary to global DIC, which assumes a continuous displacement field over the entire region of interest, local DIC treats each subset independently, producing a (dense) discrete grid of displacement vectors [47]. This makes local DIC particularly suitable to capture highly localized deformation, such as crack initiation and strain localization. Furthermore, local DIC is more robust than global DIC when the material is heterogeneous, avoiding excessive smoothing over the displacement field.

Let ${I_0}$ and *I* denote the images of the reference and deformed configurations, respectively. Under the assumption of the so-called “gray-scale conservation”, the following relation holds at any pixel position $\vec {x}$:

4 $$\begin{aligned} {I_0} (\vec {x}) \, = \, {I} (\vec {x} + \vec { u}(\vec {x})) ~, \end{aligned}$$

where $\vec {u}(\vec {x})$ is the displacement field. In practice, gray-scale conservation is never fully satisfied, even for the true displacement field, due to acquisition noise and spatial and temporal variations in lighting conditions. Therefore, the correlation residual is defined as

5 $$\begin{aligned} r(\vec {x}) \, = \, {I_0} (\vec {x}) \, - \, {I} (\vec {x} + \vec {u}(\vec {x}))~. \end{aligned}$$

Equation (5) cannot be solved for each pixel independently, as the two components of the displacement vector introduce more unknowns than the available gray scale information, making the problem underdetermined. To obtain a well-posed formulation, spatial regularization is adopted by estimating a single displacement vector over a small domain (the subset) containing multiple pixels. The correlation procedure, therefore, seeks the displacement field that minimizes the square of the residual over the considered subset domain Ω [46]:

6 $$\begin{aligned} \vec {u}(\vec {x}) = {\arg \!\min }\left\{ \int _{\Omega \subset \textrm{ZOI}} \left[ {I_0}(\vec {x}) -{I}\left( \vec {x} + \vec {u}(\vec {x}) \right) \right] ^2 \text {d} \vec {x} \right\} ~. \end{aligned}$$

In the present work, several DIC processing parameters must be selected, including the subset size, interpolation scheme, and step size.

**Table 3:** Local DIC analysis parameters used in this study

Parameter	Value
Software	MatchID
Image filtering	None
Subset size	21 px
Step size	1 px
Subset weight	Gaussian
Shape function	Affine
Matching criterion	ZNSSD
Interpolant	Bicubic spline
Displacement field filtering [52]	Gaussian
Strain window	7×7 px
Virtual strain gauge size [42]	27 px
Strain formulation	Green–Lagrange

**Fig. 8:**
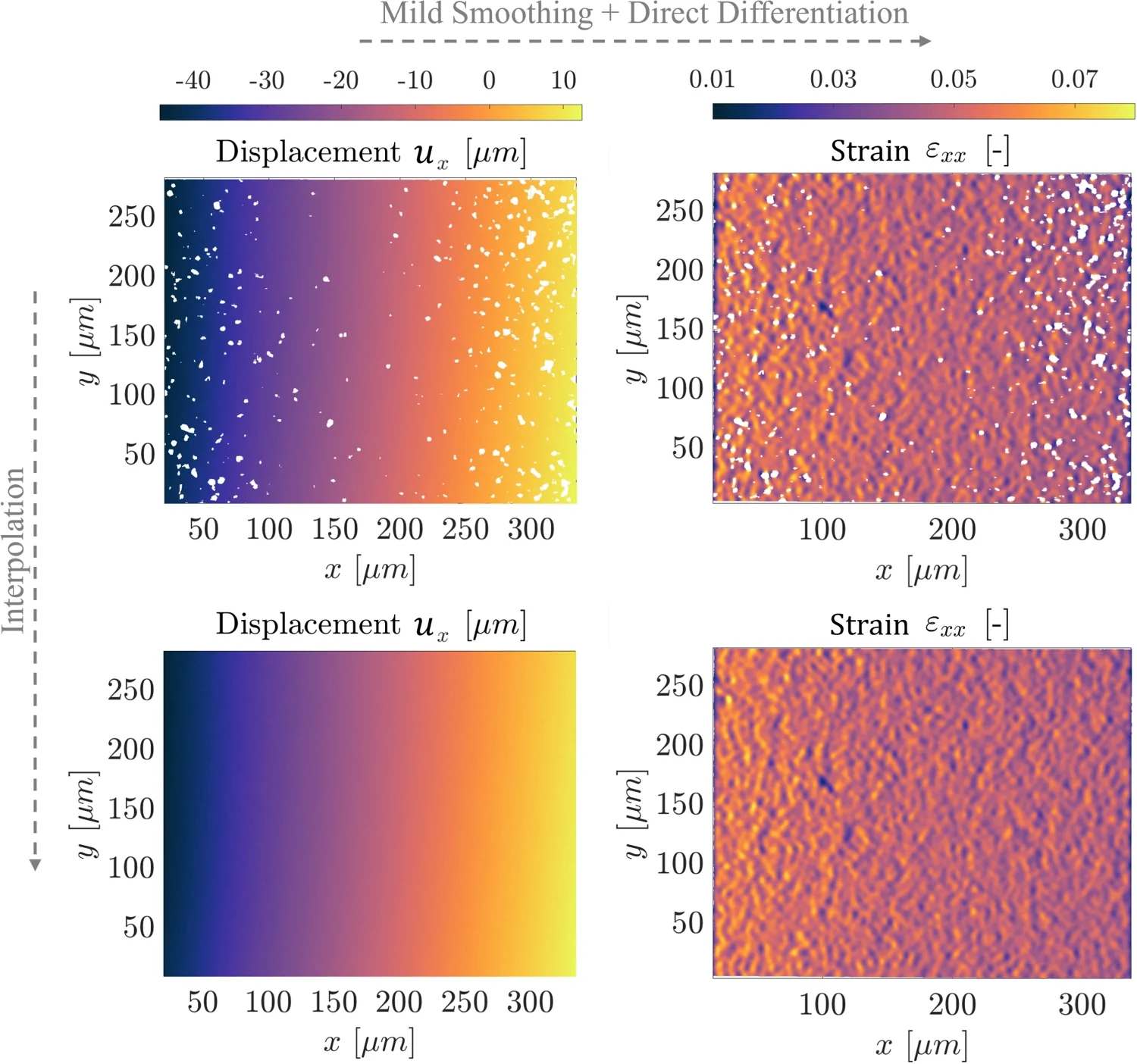
Displacement $u_x$ and strain $\varepsilon _{xx}$ maps from a representative loading step of a T-PAINT test on a Titanium White oil paint sample, obtained from the DIC and strain computation procedure based on direct differentiation. The top row shows the raw maps with missing data points; the bottom row shows the same maps after interpolation. The oscillations in the strain map arise due to mechanical heterogeneity rather than noise

Because high spatial resolution is required to capture small-scale strain localization at the microstructural level, the subset size in DIC is a critical parameter that significantly influences the accuracy, robustness, and spatial resolution of the displacement measurement. Smaller subsets provide high spatial resolution, allowing the DIC algorithm to capture fine-scale deformation details, such as strain localization. However, they contain fewer intensity information carriers (pixels), resulting in less accurate displacement measurement, especially in areas with poor speckle quality [44, 48, 49]. In contrast, larger subsets provide better signal-to-noise ratios and reduce sensitivity to image noise, brightness variations, and poor local speckle quality, thereby increasing measurement accuracy. However, this comes at the cost of a significant reduction in the spatial resolution of displacement and strain fields: larger subsets smooth out localized deformation and are therefore less suitable for resolving strain localizations [44, 48, 49]. An optimal subset size should therefore balance these competing effects.

**Fig. 9:**
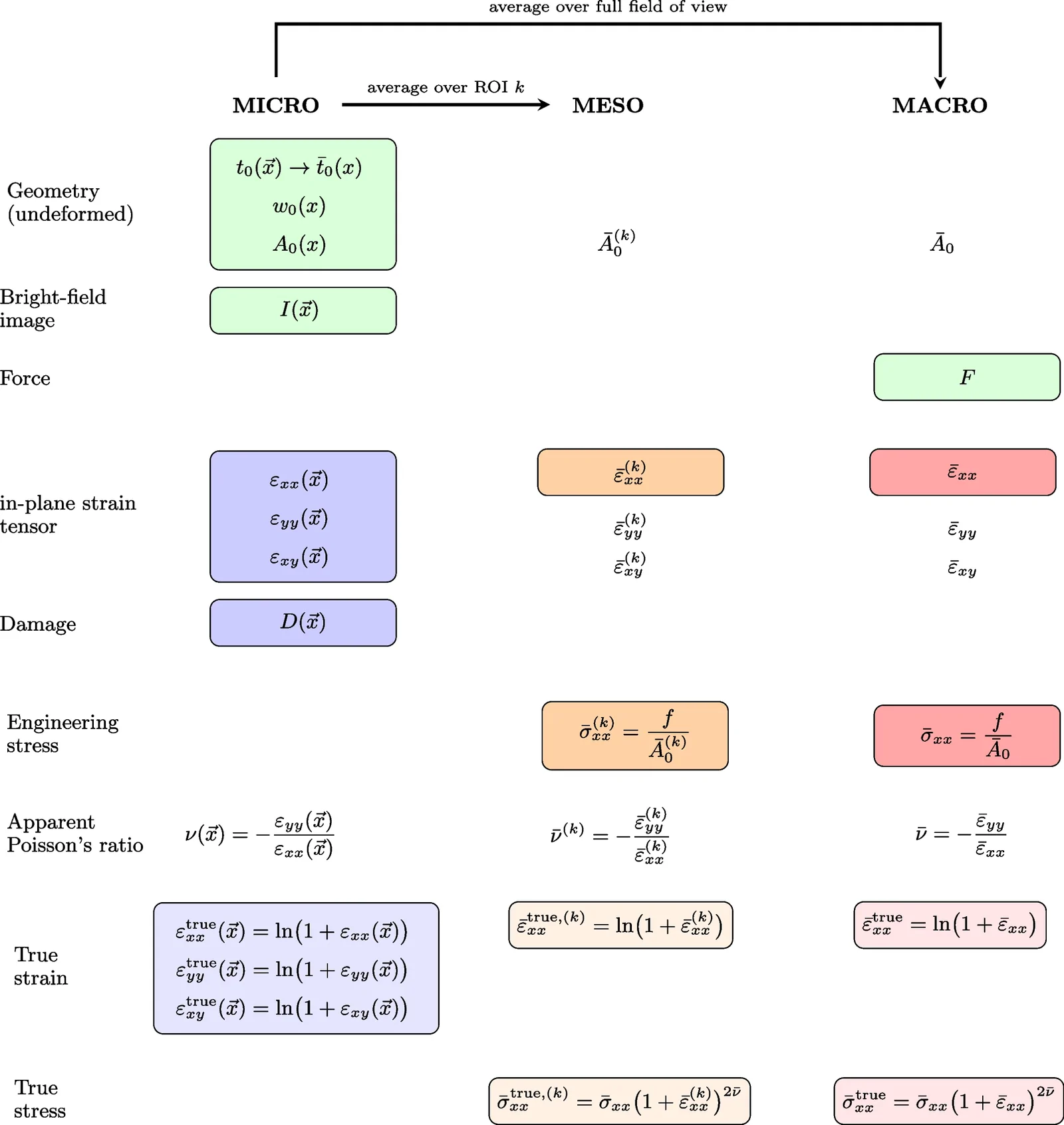
Schematic overview of the multiscale output of T-PAINT. From the acquired bright-field images, force measurements, and geometry profiles (green), the method enables the extraction of microscale DIC-based strain and damage fields (light blue), followed by the computation of mesoscale (orange) and macroscale (red) stress–strain and true stress–true strain responses

To determine the optimal interpolation strategy and subset size, a validation study was conducted using the DIC Challenge 2.0 benchmark provided by the iDIC community [50], which enables standardized assessment of spatial resolution and measurement accuracy through the Metrological Efficiency Indicator (MEI). Based on this analysis, a subset size of 21 pixels was adopted as the best compromise between accuracy and spatial resolution. Full details of the DIC Challenge validation procedure and results are reported in Appendix A: Optimization of the Displacement Field.

Moreover, a bicubic spline interpolation is applied during subset matching, a Gaussian function is used as the subset weight, and the zero-mean normalized sum of squared difference (ZNSSD) is selected as the correlation criterion. Affine deformation is adopted as the kinematic parameterization of the displacement field inside each subset.

In local DIC, the step size, defined as the distance between the centers of adjacent subsets across the region of interest, influences the density of displacement (and ultimately strain) data points. A smaller step size results in a finer measurement grid, providing higher spatial resolution in displacement and strain fields [51]. Its main drawback is increased computational time and memory usage, which is negligible in the context of the T-PAINT tests, given the limited number of high-quality images acquired. Therefore, a step size of 1 pixel is adopted.

The complete set of DIC processing parameters is summarized in Table 3.

#### Strain field computation

After acquiring the displacement data from the DIC software using the optimized settings described in Section “Theory”, the strain field is computed using a two-step procedure. First, the displacement field is smoothened using a Gaussian filter with a standard deviation of 7 pixels, implemented through the MATLAB function *nanconv* [53]. Second, the smoothed displacement components are differentiated using MATLAB’s *gradient* function, following [52], to obtain the highest possible spatial resolution in the strain field. The deformation gradient tensor is then constructed, and the Green-Lagrange strain tensor components ($\varepsilon _{xx}$, $\varepsilon _{xy}$, and $\varepsilon _{yy}$) are evaluated.

Figure 8 shows the horizontal displacement $u_x$ and the corresponding strain $\varepsilon _{xx}$ maps from a representative loading step of a T-PAINT test on a Titanium White sample. The top row displays the raw displacement and strain maps, generated without any interpolation; the bottom row presents the same fields after interpolation. This example illustrates the performance of interpolation on the displacement fields and the resulting strain maps obtained from the DIC, computed with a spatial resolution of 27 pixels (virtual strain gauge size). The oscillations in the strain map arise due to mechanical heterogeneity rather than noise.

### Summary of the Multiscale Output of the T-PAINT Method

Figure 9 summarizes the multiscale output of a T-PAINT test. The method operates on sub-millimeter paint samples and integrates geometric measurements, DIC-based evaluation of microscale strain fields, and estimation of mesoscopic and macroscopic mechanical response within a single experiment. Prior to testing, the sample geometry is quantified via high-magnification optical profilometry, providing the undeformed local thickness $\bar{t}_0(x)$ (width-averaged), width $w_0(x)$, and cross-sectional area $A_0(x)=\bar{t}_0(x)\,w_0(x)$ profile along the gauge length. During the mechanical test, at each load step, the bright-field intensity images of the sample $I(\vec {x})$ are acquired and used for full-field DIC, which provides spatially resolved displacement fields. From these, the microscale strain tensor $\boldsymbol{\varepsilon }(\vec {x})$ is obtained, capturing microstructural deformation within the paint film.

In addition, a damage field $D(\vec {x})$ can be extracted from the bright-field images, defined as $D(\vec {x}) = 1 - \frac{I(\vec {x})}{I_0(\vec {x})}$, where $I_0(\vec {x})$ denotes the reference (undeformed) intensity image. Accordingly, $D(\vec {x})=0$ corresponds to intact material, while $D(\vec {x})\rightarrow 1$ indicates severe local damage or crack initiation. Note that the intensity, damage, strain, and stress fields (introduced in the following) are all evaluated at each load step, although the explicit load-step or time dependency is omitted for conciseness. The T-PAINT method also enables the extraction of multiple mesoscale stress-strain curves from a single specimen. Along the gauge length, *N* discrete ROIs are selected at longitudinal positions $x^{(k)}$ with k=1,⋯,N. For each ROI, the axial mesoscale strain $\bar{\varepsilon }_{xx}^{(k)}$ is obtained as the ROI-average of the microscale strain, while the corresponding mesoscale stress $\bar{\sigma }_{xx}^{(k)}$ is computed from the applied (macroscopic) force F divided by the ROI-averaged cross-sectional area $\bar{A}_0\left( x^{(k)}\right)$, i.e., $\bar{\sigma }_{xx}^{(k)} = F/\bar{A}_0\left( x^{(k)}\right)$. Consequently, each ROI yields an independent stress–true strain curve provided that the mechanical response within that ROI does not influence the average response in neighboring regions. This assumption is satisfied when the ROIs are sufficiently large to average out the local kinematic heterogeneity visible in the strain field. For this reason, a relatively low number of ROIs (e.g., 5) are selected across the field of view. The resulting ensemble of mesoscale stress-strain curves $\bigl [\sigma _{xx}^{(k)},\;\varepsilon _{xx}^{(k)}\bigr ]_{k=1}^{N}$ offers therefore a meaningful representation of the statistical variation of the material behavior, which is particularly valuable in cultural heritage applications where only very limited amounts of original paint can be extracted for testing.

The macroscopic mechanical behavior is obtained by averaging the axial strain component $\varepsilon _{xx}(\vec {x})$ over the full field of view to obtain the macroscopic strain $\bar{\varepsilon }_{xx}$. From the applied force *F* and the average cross-sectional area $\bar{A}_0$ in the undeformed configuration, the macroscopic stress follows as $\bar{\sigma }_{xx}=F/\bar{A}_0$. The corresponding macroscopic stress–strain curve provides global properties such as the Young’s modulus $\bar{E}$, yield strength, ultimate tensile strength $\bar{\sigma }_\textrm{UTS}$, and strain at break.

Finally, a true stress-true strain response can also be derived, which provides a more physically representative description of the visco-elasto-plastic material deformation. The global axial true strain can be calculated as $\bar{\varepsilon }_{xx}^{true} \,= \ln (1+ \bar{\varepsilon }_{xx})$ [54]. Assuming an approximately isotropic material response at the macroscopic scale, an effective Poisson ratio can be estimated from the global transverse strain component as $\bar{\nu } = -\bar{\varepsilon }_{yy}/\bar{\varepsilon }_{xx}$, where $\bar{\varepsilon }_{yy}$ is obtained as the spatial average of the microscopic field ${\varepsilon }_{yy}(\vec {x})$. The true stress can be thus derived as $\bar{\sigma }_{xx}^{true}\,=\bar{\sigma }_{xx}(1+\bar{\varepsilon }_{xx})^{2\bar{\nu }}$. The same procedure can be applied at the ROI level to obtain true-stress–true-strain curves for each mesoscale region.

**Fig. 10:**
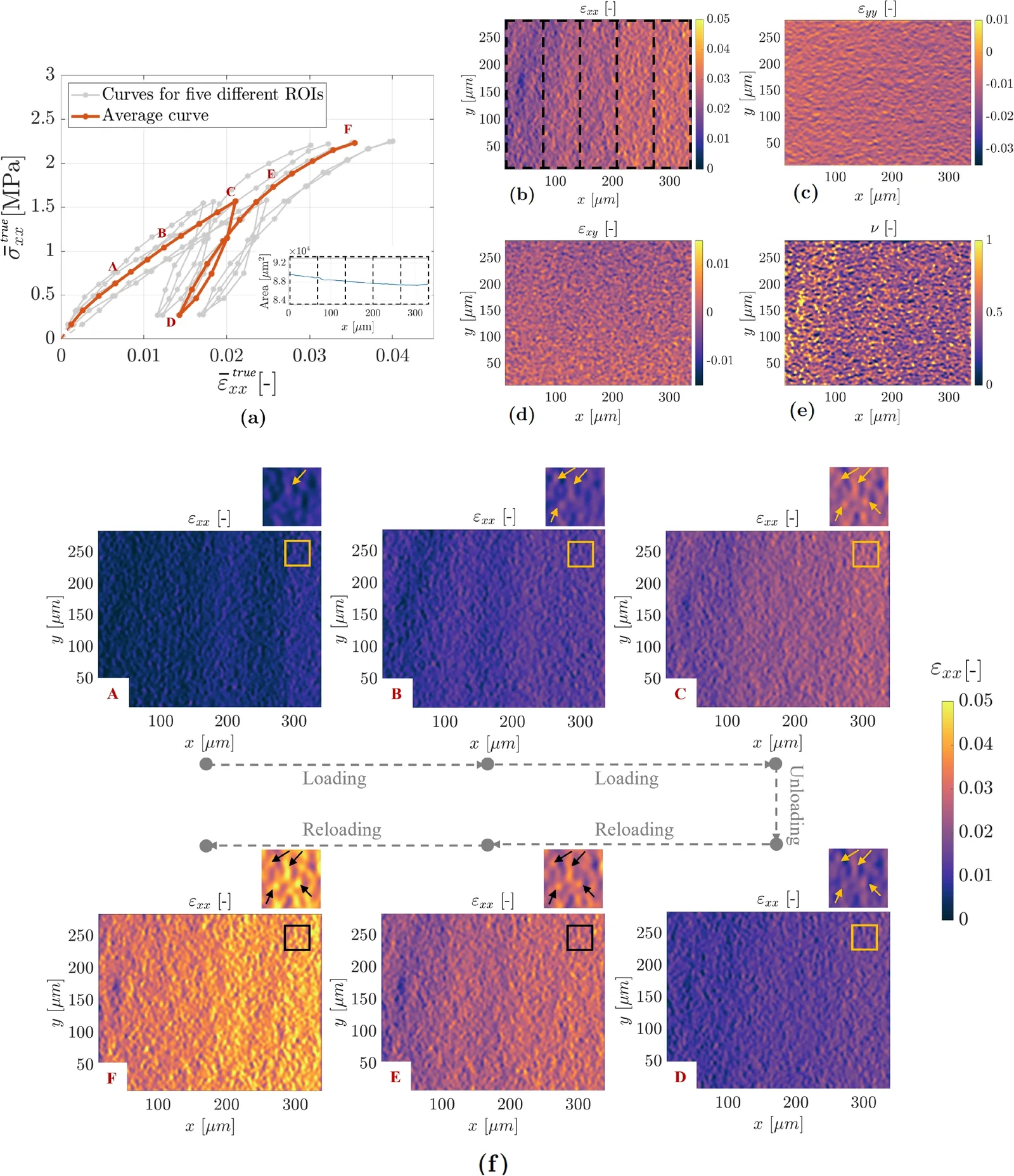
Overview of the multiscale data obtained from a single T-PAINT test on a 14-weeks-aged Titanium White oil paint sample. (**a**) Global true stress–true strain curve during loading, unloading, and reloading. Grey lines represent mesoscale responses evaluated at 5 different ROIs; the inset shows the initial cross-section with ROI locations. (**b-e**) Full-field maps of the strain components $\varepsilon _{xx}$, $\varepsilon _{yy}$, $\varepsilon _{xy}$, and apparent Poisson’s ratio $\nu = -\varepsilon _{yy}/\varepsilon _{xx}$ at loading step E. (**f**) Evolution of the $\varepsilon _{xx}$ field during loading, unloading, and reloading (points A–F in (**a**)). All strain fields are shown in the reference configuration

## Proof of Principle of the Methodology Performance

**Fig. 11:**
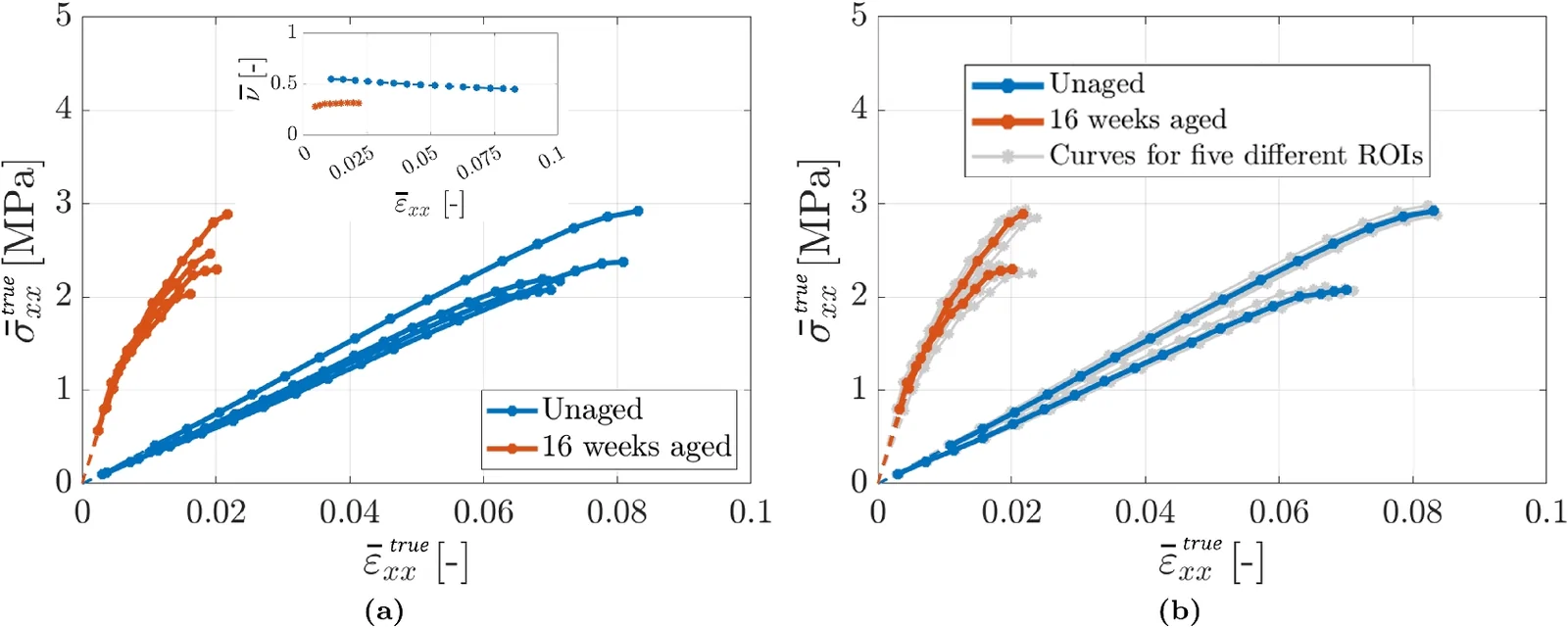
True stress–true strain curves obtained from unaged (blue color) and aged (red color) Titanium White samples. (**a**) Global responses; inset: global Poisson’s ratio $\bar{\nu }$ extracted from $\bar{\varepsilon }_{xx}$ and $\bar{\varepsilon }_{yy}$. (**b**) Mesoscale curves based on 5 ROI data (grey color) and global curves for two T-PAINT tests on unaged and aged samples

### Full Demonstration on a Titanium White Sample

A loading–unloading experiment performed on a 14-week-aged Titanium White oil paint sample is discussed here to demonstrate the full multiscale capability of the T-PAINT methodology. Figure 10(a) shows the macroscopic true stress–true strain curve (orange color), obtained from the micro-tensile experiment, together with five mesoscale true stress–strain curves (grey color) extracted from local measurements along the specimen length.

The global strain is obtained by averaging the microscale axial strain component computed via DIC over the full field of view, while the global stress follows from combining the applied force with the average cross-sectional area measured prior to testing through high-magnification optical profilometry, as described in Section “Dimension Measurements”. The inset in Fig. 10(a) precisely shows the local cross-sectional area along the specimen.

The mesoscale stress-strain curves are evaluated in five individual ROIs, indicated by dashed vertical lines both in the area inset and in the strain map in Fig. 10(b). The axial strain obtained from DIC is averaged over each ROI, and the corresponding local stress is computed from the applied force divided by the ROI-averaged cross-sectional area. These mesoscale responses clearly show how variations in local cross-section and morphology translate into different apparent mechanical responses along the specimen.

All stress-strain curves (macro and meso) are corrected for the small residual stress generated prior to the onset of loading. The initial, dashed portion of each curve is an extrapolation to the origin, constructed by fitting a second-order polynomial through the data points (loading portion). The gradually decreasing slope with increasing strain indicates the development of inelastic deformation within the paint. After unloading, a permanent deformation remains, consistent with a visco-plastic response of the polymeric network. Upon reloading, the slope of the curve evolves back approximately toward the original loading trend until brittle failure.

From the macroscopic stress–strain curve, global mechanical properties are extracted. The Young’s modulus $\bar{E}$ is obtained from the initial slope of the curve, while the strength $\bar{\sigma }_\textrm{UTS}$ is computed from the maximum applied force divided by the minimum measured cross-sectional area.

For this sample, the Young’s modulus is approximately 150 MPa with a standard deviation of 28 MPa, the ultimate tensile strength is 2.23 MPa with a standard deviation of 0.022 MPa, and the strain at break is 0.035 with a standard deviation of 0.004. The standard deviations are calculated from the ensemble of mesoscale curves.

At the microscale, full-field DIC provides spatially resolved maps of the strain tensor components $\varepsilon _{xx}$, $\varepsilon _{yy}$, $\varepsilon _{xy}$ and the apparent Poisson ratio ν at each loading step. Figure 10(b–e) show these fields at a selected loading step (marked as E in the global stress-strain curve). The evolution of the axial strain field during loading, unloading, and reloading is shown in Fig. 10(f). As highlighted in the zoomed regions above the strain maps, small strain localization regions emerge during loading, decrease during unloading, and reappear at the same locations upon reloading. The consistency in this mechanism confirms that these features originate from underlying microstructural mechanisms rather than noise. Possibly, they originate from stiff pigment inclusions that constrain deformation in their immediate vicinity.

**Fig. 12:**
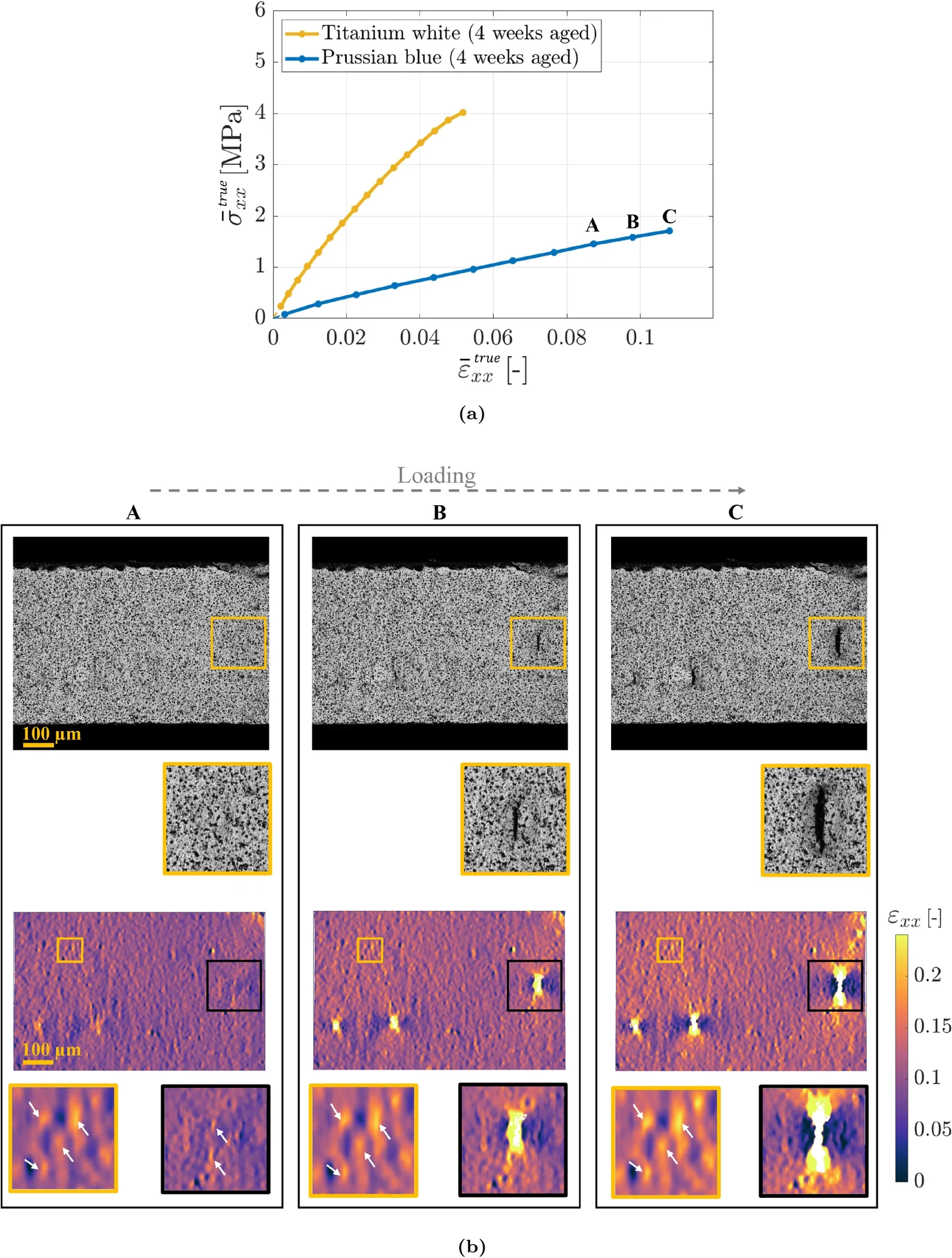
(**a**) Global responses for 4-weeks-aged Titanium White and Prussian Blue oil paint samples. (**b**) Evolution of bright-field intensity images (top row) and corresponding $\varepsilon _{xx}$ strain maps (bottom row) for the 4-weeks-aged Prussian Blue oil paint sample. The zooms highlight the formation and growth of strain localizations, which eventually coalesce and lead to cracking. All strain maps are shown in the reference configuration

### Applicability to Paint Samples with Different Aging Conditions

The applicability of the T-PAINT methodology to materials with significantly different mechanical properties is next assessed by testing four Titanium White oil paint samples in unaged condition and four after artificial aging (16 weeks) - see Table 2. Figure 11(a) summarizes the global true stress–true strain curves for all specimens, with blue indicating unaged samples and orange the aged ones. The inset in Fig. 11(a) additionally reports the corresponding global Poisson’s ratio $\bar{\nu }$.

The evolution of the macroscopic behavior with aging is clearly visible. Unaged samples exhibit a more compliant and more ductile response, whereas aged samples become progressively stiffer and more brittle. These trends are consistent with macroscopic measurements reported in the literature, for example [27] for Titanium White and [15] for Lead White oil paint.

From the global stress–strain curves, mechanical properties are extracted. The Young’s modulus increases from approximately 33 MPa (standard deviation of  3 MPa) in the unaged condition to about 270 MPa (standard deviation of 19 MPa) after 16 weeks of aging, while the strain at break decreases from 0.077 (standard deviation of 0.006) to 0.019 (standard deviation of 0.0025). The ultimate tensile strength values for unaged and 16-weeks-aged samples do not vary significantly and are 2.4 MPa (standard deviation 0.36 MPa) and 2.5 MPa (standard deviation 0.36 MPa), respectively. The elastic moduli of the unaged samples fall within the same order of magnitude as those reported by [27], although slightly higher. While these values are not directly comparable (due to differences in pigment volume concentration, aging protocol, and other material factors), the discrepancy may originate from the use of local, DIC-based strain measurements in the present study, in contrast to the global strain evaluation used in [27], which tends to overestimate strain and thus underestimate the initial modulus.

The extracted Poisson’s ratio also reflects the influence of aging: unaged samples display a response close to incompressible behavior, whereas aged specimens show a reduced average Poisson’s ratio of approximately $\bar{\nu } \approx {0.27}$.

The mesoscale analysis is consistent with these global trends. Figure 11(b) shows the ROI-based responses for two T-PAINT tests per aging condition. The spread among the ROI curves within these two single specimen sets (per aging type) is comparable to the variability observed across independent samples. For applications to historical materials, where available material is extremely limited, this emphasises an important advantage: a small number of T-PAINT tests can provide statistical insight normally requiring multiple conventional tensile tests, with a minimally invasive type of testing.

### Applicability to Different Paint Formulations and Microscale Damage

The applicability of the T-PAINT methodology is finally demonstrated on oil paint systems with different chemical compositions. Figure 12(a) compares the global true stress–true strain curves of a 4-weeks-aged Prussian Blue oil paint sample (blue color) with a 4-weeks-aged Titanium White oil paint sample (yellow color). Prussian Blue exhibits a more compliant and ductile mechanical behavior compared to Titanium White. For the tested Prussian Blue sample, the Young’s modulus is approximately 26 MPa, the ultimate tensile strength is about 1.6 MPa, and the strain at break is 0.124. For the 4-week aged Titanium White sample, the Young’s modulus is 118 MPa, the ultimate tensile strength is 4 MPa, and the strain at break is 0.052.

The microscale strain fields provide insight into the mechanisms underlying the relatively more ductile response of Prussian Blue paint, in contrast to the more brittle behavior observed for Titanium White paint (which is comparable to the microstructural images and strain map shown in Fig. 7). Figure 12(b) illustrates the evolution of the axial strain component $\varepsilon _{xx}$ during loading for the Prussian Blue sample, at the last three loading steps (A–C) on the global curve, together with the corresponding bright-field images. Distinct strain localization regions consistently appear on the two sides of stiff pigment agglomerates. As the applied tensile load increases, these localization bands grow and gradually coalesce, eventually leading to crack nucleation (damage D=1) at their intersection. This mechanism is particularly clear in the zoomed regions in Fig. 12(b).

## Conclusions

This paper presents T-PAINT, a novel experimental methodology to characterize the mechanical response of small-sized oil paint samples, through *in-situ* micro-tensile testing combined with optical profilometry and full-field Digital Image Correlation. The method relies on accurate cross-sectional measurement based on capturing height profiles of both the top and bottom surfaces of the samples. From bright-field images acquired during loading, DIC is applied to obtain high-resolution displacement, strain, and damage fields. Depending on the sample, either the natural surface texture or a tailored 3D speckle pattern is used to obtain a reliable DIC correlation.

The resulting field measurements enable the construction of multiple true stress–true strain curves and the extraction of mesoscopic and macroscopic mechanical properties (including Young’s modulus, yield strength, ultimate tensile strength, strain at break, and Poisson’s ratio) from a single experiment. The method is applicable to oil paints with different aging conditions and chemical compositions. A proof of principle of the method is demonstrated on different paint samples, showing that:

- Microscale field measurements provide insight into the deformation and failure mechanisms of oil paint. Strain localization is consistently observed near pigment-rich regions, where damage accumulates and microcracks initiate. These local mechanisms correlate directly with the mesoscale and macroscopic stress–strain responses extracted from the same experiments.
- Aged samples consistently exhibit increased stiffness and brittleness, whereas differences in pigment formulation (Prussian Blue versus Titanium White) lead to clearly different ductile or brittle failure responses. These trends agree with published macroscopic data and demonstrate that T-PAINT can reliably capture the mechanical consequences of both chemical formulation and aging.
- The true stress–true strain curves obtained from multiple ROIs within a single specimen exhibit a variability comparable to that obtained from the testing of several independent samples. This enables statistically relevant mechanical characterization on a limited number of small samples, which is an essential advantage when analyzing original historical oil paints, where the number of available samples is often limited.

The proposed methodology also creates opportunities for future developments. Integrating the mechanical characterization with spectroscopic techniques such as FTIR would enable the direct coupling of local chemistry and mechanical response, allowing us to correlate aging-related chemical changes in the binder and pigment–binder interface with variations in stiffness, ductility, and fracture mechanisms. In addition, the methodology could be extended to micro-tensile testing at even smaller length scales, including *in-situ* scanning electron microscope (SEM) experiments at very high magnification, which enable detailed investigation of how local material inhomogeneities influence material behaviour. Establishing these quantitative chemo–mechanical relationships will be an important step toward predictive models of the long-term structural behavior of historical paint films.

## Figures and Tables

**Fig. 13:**
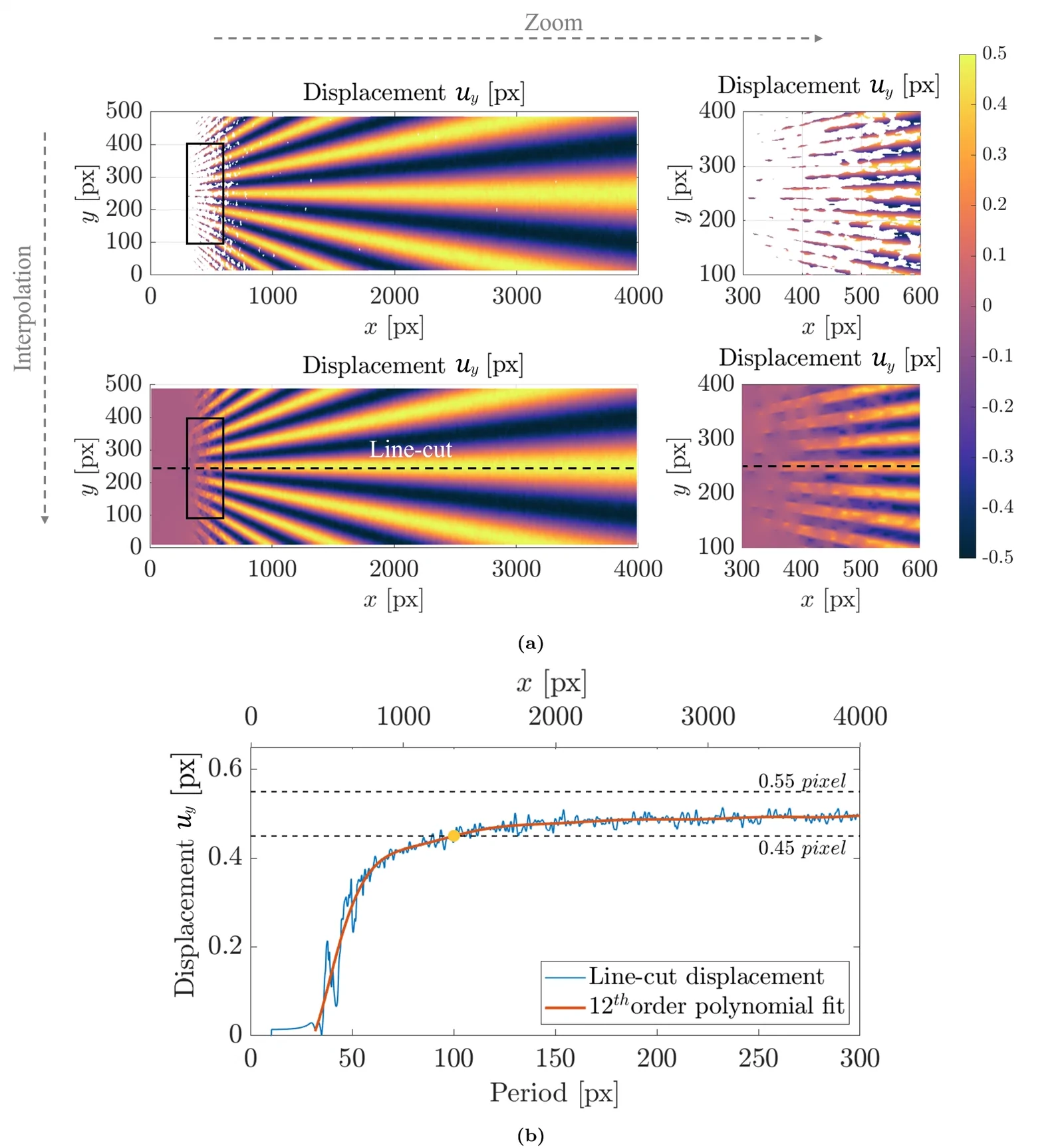
(**a**) Vertical displacement field $u_y$ obtained from the deformed image in the DIC Challenge 2.0 using the *Star 5* image set with a subset size of 21 pixels. The top-left panel shows the displacement field with missing data points, and the bottom-left panel shows the same field after interpolation using *Method 4*. Zoomed-in views of a selected region are shown on the right to highlight the missing data points. (**b**) Vertical displacement $u_y$ along the line-cut obtained with a subset size of 21 pixels

**Fig. 14:**
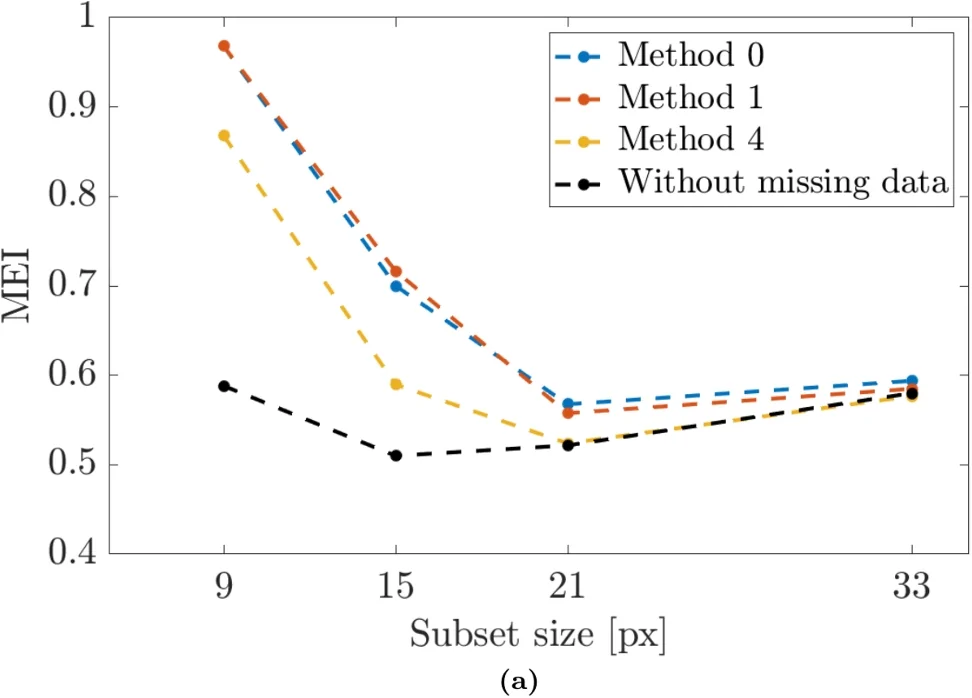
MEI values as a function of subset size for three different interpolation methods (*Method 0*, *Method 1*, and *Method 4*). The black data points correspond to the case without missing data and are included for comparison

**Fig. 15:**
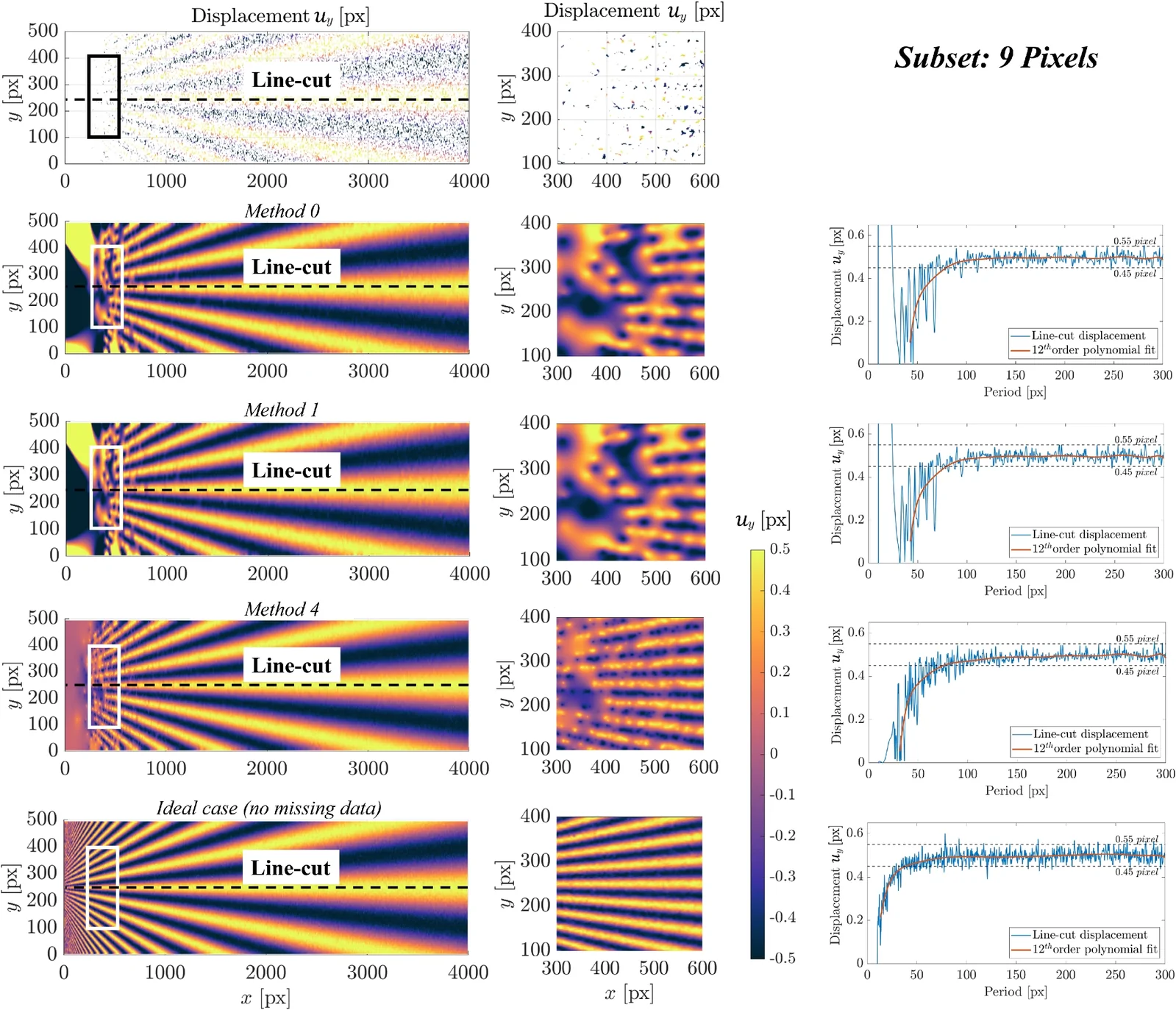
Displacement fields results for the deformed image, as a result of the “DIC Challenge 2.0” applied on *Star 5* images using subset size of 9 pixels. The top-left figure shows the resulting displacement field containing missing data points, while the remaining figures on the left display the same field after interpolation using the different methods. The ideal case without missing data is also depicted at the bottom for comparison. Zoomed versions of the displacement fields at a particular region are included to highlight the missing data points. The corresponding vertical displacement versus period curves along the line-cut for each displacement field are presented on the right

**Fig. 16:**
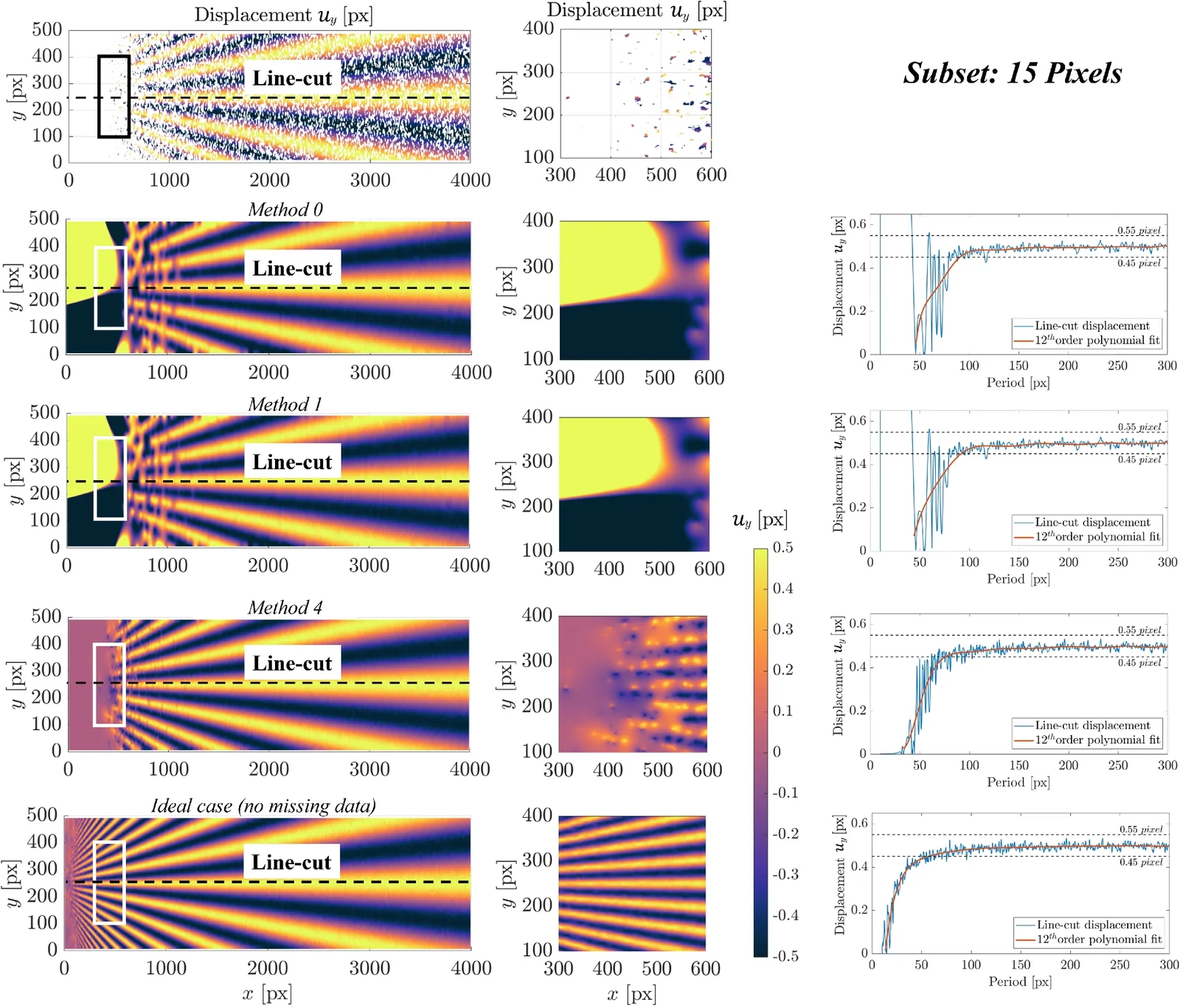
Displacement fields results for the deformed image, as a result of the “DIC Challenge 2.0” applied on *Star 5* images using a subset size of 15 pixels. The top-left figure shows the resulting displacement field containing missing data points, while the remaining figures on the left display the same field after interpolation using the different methods. The ideal case without missing data is also depicted at the bottom for comparison. Zoomed versions of the displacement fields at a particular region are included to highlight the missing data points. The corresponding vertical displacement versus period curves along the line-cut for each displacement field are presented on the right

**Fig. 17:**
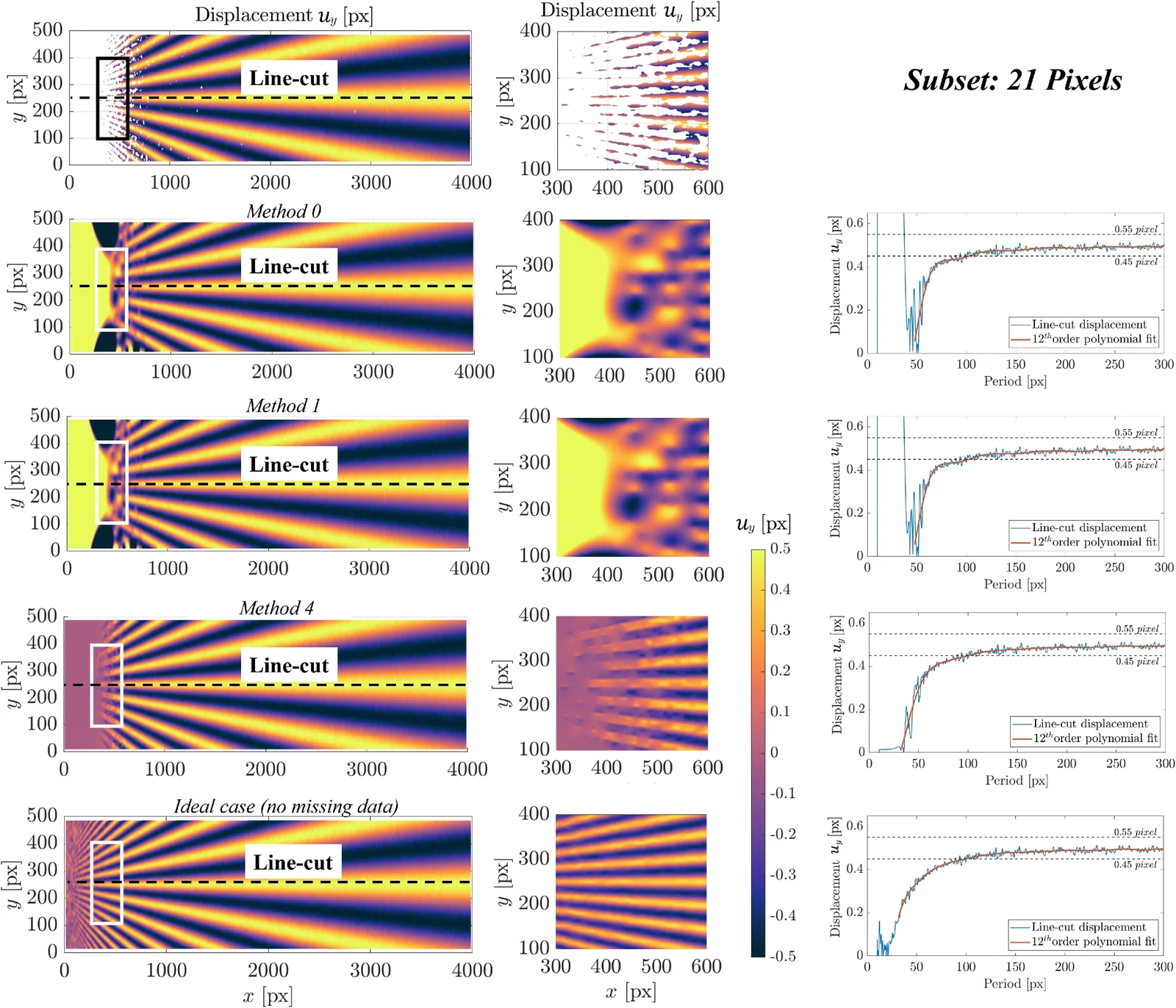
Displacement fields results for the deformed image, as a result of the “DIC Challenge 2.0” applied on *Star 5* images using a subset size of 21 pixels. The top-left figure shows the resulting displacement field containing missing data points, while the remaining figures on the left display the same field after interpolation using the different methods. The ideal case without missing data is also depicted at the bottom for comparison. Zoomed versions of the displacement fields at a particular region are included to highlight the missing data points. The corresponding vertical displacement versus period curves along the line-cut for each displacement field are presented on the right

**Fig. 18:**
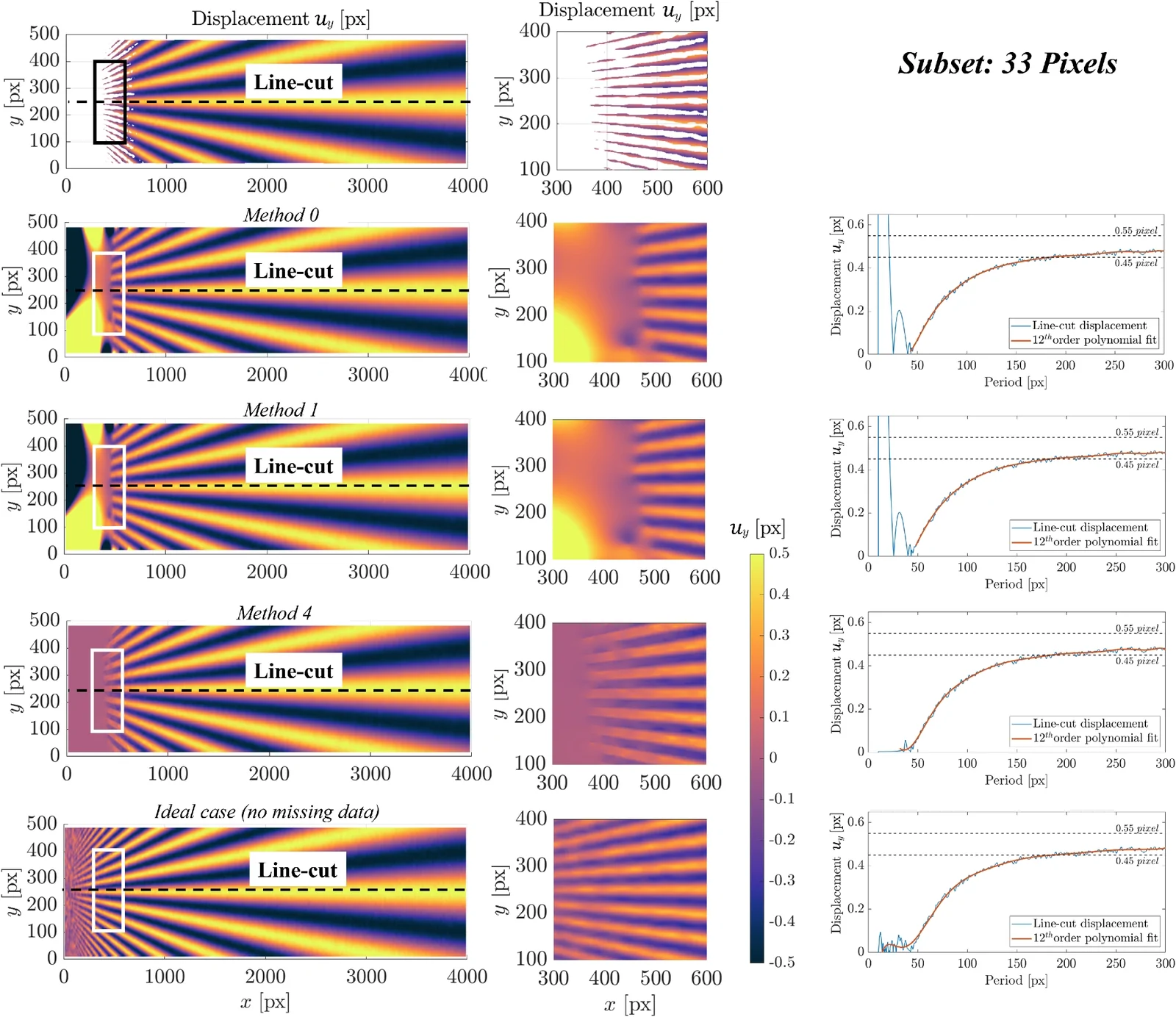
Displacement fields results for the deformed image, as a result of the “DIC Challenge 2.0” applied on *Star 5* images using a subset size of 33 pixels. The top-left figure shows the resulting displacement field containing missing data points, while the remaining figures on the left display the same field after interpolation using the different methods. The ideal case without missing data is also depicted at the bottom for comparison. Zoomed versions of the displacement fields at a particular region are included to highlight the missing data points. The corresponding vertical displacement versus period curves along the line-cut for each displacement field are presented on the right

## Data Availability

Data will be available upon reasonable request.

## Appendix A: Optimization of the Displacement Field

Although both natural and externally applied speckle patterns yield good image contrast, some small regions may still lack sufficient pattern quality, leading to missing data points in the displacement fields, particularly when high-resolution results are desired. The subset size must therefore be chosen to maximize spatial resolution while avoiding an excessive number of missing subsets, which would make interpolation between properly correlated subsets unreliable.

To assess the interpolation strategy used to fill missing data, a validation exercise is performed based on the DIC Challenge 2.0 benchmark provided by the iDIC community [50]. The DIC Challenge 2.0 framework is designed to assess the accuracy and spatial resolution of 2D-DIC implementations by introducing standardized image sets and evaluation metrics, including the Metrological Efficiency Indicator (MEI). Building on the first DIC Challenge [55], this second challenge focuses on more rigorous quantification of spatial resolution in 2D-DIC codes.

In this work, the *Star 5* image set is used. It contains high-resolution noisy images with known displacement. The set consists of three images: a noisy reference image, an undeformed noise floor image, and a deformed noisy image in which a true ±0.5 pixel sinusoidal vertical displacement field $u_y$ is applied. The resulting displacement period varies linearly from 10 pixels to 300 pixels across the width of the image.

Performing DIC analysis on these images provides two displacement fields: i) the displacement field derived from the correlation of the noisy reference image and the undeformed noise floor image whose center row (referred to as a line-cut) is used to compute the measurement resolution in the absence of motion, defined as the standard deviation (n=1σ) of the displacement measurement resolution; ii) the displacement field derived from the correlation of the noisy reference image and the deformed noisy image whose line-cut is used to evaluate the spatial resolution, expressed as a cutoff period. Following the DIC Challenge 2.0 guidelines, spatial resolution is defined as the period in which the recovered displacement signal exhibits a 10% attenuation, indicated as $l_{10\%}$ [50]. The Metrological Efficiency Indicator (MEI) can then be determined as

7 $$\begin{aligned} {\textrm{MEI}} = n ~ l_{10\%} ~, \end{aligned}$$

where lower MEI values indicate better DIC performance.

After obtaining the displacement field from DIC, missing data points are artificially introduced. To interpolate the missing data points, the MATLAB function $inpaint\_nans$ is integrated into the main code [56]. This function fills NaN values in a 2D array using surrounding non-NaN elements and offers six different interpolation schemes (labeled *Method 0* to *Method 5*). Each method is evaluated independently by computing the measurement resolution, spatial resolution, and the corresponding MEI. Moreover, different subset sizes are tested, providing insight into how subset size influences the trade-off between measurement density (i.e., missing data) and overall DIC performance.

Figure 13(a) presents an example of the vertical displacement fields $u_y$ resulting from the correlation of the noisy reference image with the deformed noisy image. The top left panel shows the displacement field with missing data points, while the bottom left panel shows the same field after interpolation using *Method 4*. The zoomed-in views at the right highlight the missing and interpolated regions. The black dashed line indicates the line-cut used to calculate the measurement and spatial resolutions. The corresponding vertical displacement along the line-cut is depicted in Fig. 13(b), showing the variation in displacement amplitude as a function of period across the image width. Following the DIC Challenge 2.0 guidelines, a 12*th* order polynomial is fit to the displacement-period data. The 10% attenuation level of the signal (horizontal dashed line of 0.45 pixel) is further used to determine the spatial resolution, which is approximately equal to 100 pixels. The measurement resolution, obtained by correlating the undeformed noise floor image with the noisy reference image, is calculated as the standard deviation of the displacement along the same line-cut and is equal to 0.0052 pixels. The MEI value is determined using Equation (7), resulting in a value of 0.52 [50].

Among the six interpolation methods available, three were discarded after preliminary testing on the strain maps because they produced comparatively less accurate results. Figure 14 summarizes the MEI values obtained for the remaining three interpolation methods (*Method 0*, *Method 1* and *Method 4*) as a function of subset size. *Method 4* consistently provides the lowest MEI values and therefore exhibits the best overall performance. *Method 4* is based on a spring analogy formulation, where each node is connected to its horizontal, vertical, and diagonal neighbors by virtual springs with a nominal length of zero, resulting in a smooth displacement field without erratic interpolation-induced displacement peaks [56]. The results in Fig. 14 additionally show that the subset sizes of 15, 21, and 33 pixels (using *Method 4*) yield MEI values that fall within the range reported for local DIC methods with affine kinematics in [50] and are comparable to those obtained without missing data. Among the different considered sizes, a subset size of 21 pixels provides the best compromise between accuracy and spatial resolution. A subset size of 33 pixels reduces spatial resolution, while a subset size of 15 pixels does not contain sufficient intensity information, which affects accuracy. A subset size of 21 pixels is therefore selected in this work. This size is also commonly used in commercial DIC software packages.

The MEI-based comparison above provides the quantitative basis for selecting the interpolation strategy and subset size adopted in this study. For completeness and reproducibility, the full displacement fields and corresponding line-cut responses obtained from the DIC Challenge 2.0 benchmark are additionally reported below for all tested subset sizes and interpolation methods. Figures 15, 16, 17 and 18 show the detailed results obtained for subset sizes of 9, 15, 21, and 33 pixels, respectively. These figures confirm the trends already identified from the MEI analysis, for which increasing subset size reduces measurement scatter but degrades spatial resolution, while *Method 4* provides the most stable interpolation performance.

## Footnotes

1 Note that here the term *in-situ* is used in the sense commonly employed in experimental mechanics, referring to tests performed within an observation instrument rather than to direct measurements on the painting itself, as the term would generally imply in the cultural heritage community.
